# A de novo designed coiled coil-based switch regulates the microtubule motor kinesin-1

**DOI:** 10.1038/s41589-024-01640-2

**Published:** 2024-06-07

**Authors:** Jessica A. Cross, William M. Dawson, Shivam R. Shukla, Johannes F. Weijman, Judith Mantell, Mark P. Dodding, Derek N. Woolfson

**Affiliations:** 1 School of Biochemistry, University of Bristol, Bristol, UK; 2 School of Chemistry, University of Bristol, Bristol, UK; 3 Bristol BioDesign Institute, University of Bristol, Bristol, UK

**Keywords:** Protein design, Enzyme mechanisms

## Abstract

Many enzymes are allosterically regulated via conformational change; however, our ability to manipulate these structural changes and control function is limited. Here we install a conformational switch for allosteric activation into the kinesin-1 microtubule motor in vitro and in cells. Kinesin-1 is a heterotetramer that accesses open active and closed autoinhibited states. The equilibrium between these states centers on a flexible elbow within a complex coiled-coil architecture. We target the elbow to engineer a closed state that can be opened with a de novo designed peptide. The alternative states are modeled computationally and confirmed by biophysical measurements and electron microscopy. In cells, peptide-driven activation increases kinesin transport, demonstrating a primary role for conformational switching in regulating motor activity. The designs are enabled by our understanding of ubiquitous coiled-coil structures, opening possibilities for controlling other protein activities.

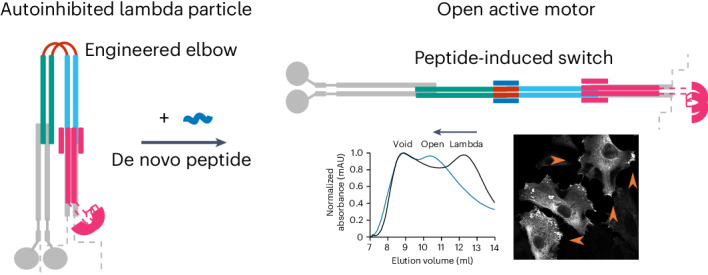

## Abstract (web summary)

The kinesin-1 motor protein accesses open active and closed autoinhibited states. These states are regulated by a flexible elbow within a complex coiled-coil architecture. Now, a conformational switch has been developed by engineering the elbow to create a closed state that can be controllably opened with a de novo designed peptide to increase kinesin transport inside cells.

## Main

Conformational switches are key regulators of protein function and activity. Specifically, allostery refers to regulation of active site function through a conformational change in a protein or protein complex brought about by binding an effector molecule to alter the protein’s function or activity. An ability to engineer allostery into natural and de novo designed proteins would have considerable impact in cell, chemical and synthetic biology. However, amongst other challenges, allosteric binding sites are often distant from the active sites of enzymes. This makes coupling the two sites through conformational switches to render successful designs a complex endeavor.

The predominant conformation of a protein is determined by its lowest energy state for a given set of conditions. Conformational switching requires delicate control of the equilibrium between two or more states, with fine-tuned energetics to drive exchange in response to a stimulus. Although protein design is advancing rapidly^1–3^, capturing such balances in de novo proteins is extremely challenging. Indeed, protein design has largely sought to maximize the free energy difference between a single, desired, folded structure and the unfolded and alternative states. In this respect, the state of the art of protein design is impressive, as defined single states are increasingly being delivered with high accuracy^4–7^. However, many of these are hyperstable^8–11^, probably less dynamic than natural protein structures, and thus are limited for exploring conformational dynamics, alternative states and allostery. Therefore, the design of more plastic and potentially switchable protein structures is a priority. Indeed, the field has sought to mimic protein conformational switches through multistate design for some time^3,12,13^; and there are examples of allosteric switches that have been engineered into natural proteins^14^, or designed de novo^15–19^. Another challenge is to apply developments from largely test tube-based designs to manipulate complex natural proteins in situ.

Dynamics between different conformational states are particularly important in cytoskeletal motor proteins, which are involved in a range of cellular processes, including intracellular transport, cell division and cell migration. Motor activity and regulation are critical for healthy cell function, and dysregulation can lead to disease^20–22^. Therefore, strategies to manipulate motor protein activity would progress fundamental cell biology and biomedical applications. A key aspect of motor regulation involves autoinhibitory interactions within the multidomain/multisubunit protein complexes.

**Fig. 1:**
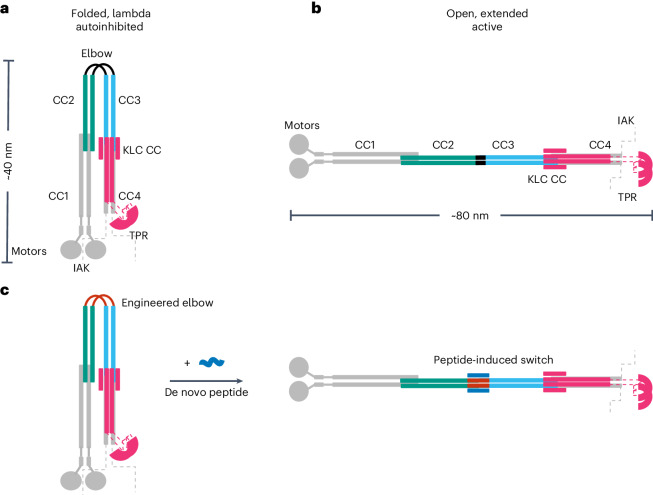
Architecture of the autoinhibited and active states of kinesin-1 and target design. **a**,**b**, Schematic illustrating the autoinhibited lambda particle (**a**) and open conformer (**b**) of heterotetrameric kinesin-1. Globular (motor and tetratricopeptide repeat (TPR)) domains are labeled and the coiled-coil domains of the KHCs are numbered CC1–CC4, as previously described^28^. KHCs are shown with motor domains (motors) and coiled-coil domains 1 and 4 (CC1 and CC4) in gray. Gray dashed lines indicate the unstructured KHC C-terminal tails that contain the IAK regulatory motif. The key coiled-coil domains discussed in this study are highlighted in teal (CC2) and cyan (CC3). The elbow between CC2 and CC3 is in black. KLCs are shown in magenta, indicating the six-helix bundle predicted at the KHC–KLC^28^ interface, with dashed lines indicating the unstructured linker to the globular TPR domains involved in cargo recognition. **c**, The aim of this work is to engineer a de novo peptide (red) into the elbow of a lambda state to construct a peptide-inducible (blue) conformational switch to the open state.

For example, kinesin-1 is a ubiquitous microtubule motor involved in intracellular cargo transport within most eukaryotic cells, with a prominent role in axonal transport. It forms a heterotetramer comprising two motor-bearing heavy chains (KHCs) and two cargo-binding/regulatory light chains (KLCs)^23^. When not transporting cargo, kinesin-1 enzymatic activity is inhibited by interactions between C*-*terminal regulatory motifs (isoleucine-alanine-lysine (IAK) motifs) and the N*-*terminal motor domains of the KHCs (Fig. 1a). Effectively, these interactions cross-link the motor domains, suppressing microtubule-dependent adenosine triphosphatase (ATPase) activity^24–27^. Recently, we combined computational structure prediction with single-particle negative-stain electron microscopy (NS-EM) to visualize the complex coiled-coil architecture of the kinesin-1 autoinhibited state. This revealed a flexible hinge between two coiled-coil domains (CC2 and CC3) of KHC, ‘the elbow’. This region is critical for the complex to switch between an open state (Fig. 1b) and a folded-over ‘lambda’ particle, which brings the IAK motifs and motor domains together (Fig. 1a)^28^. This is supported by experiments combining cross-linking and mass spectrometry^29^.

It is generally thought that the open form and the lambda particle are associated with the active and autoinhibited states of kinesin-1, respectively, but the extent of the conformational change and how it is regulated is unclear. We reasoned that this could be addressed through protein design to target the coiled coil-based switch. Coiled coils are attractive targets because the unprecedented understanding of them provides a strong basis for design^30^. Indeed, the de novo design of static coiled coils with defined oligomeric states is considered largely solved^1,30^. Remaining challenges in coiled-coil design include making coiled coil-based switches^31–36^ and using our understanding to intervene in biological processes directly in situ.

Here we address these questions in kinesin cell biology and challenges in protein design to deliver de novo protein–peptide interactions that control the kinesin-1 motor in cells. To do this, we apply protein design principles to re-engineer the motor-activation switch and render it activatable by a de novo designed cell-penetrating peptide (Fig. 1c). This establishes a clear role for a large-scale conformational change pivoting on the elbow to regulate motor activity, and it demonstrates the potential to manipulate coiled-coil switches in biology to understand and hijack protein function.

## Results

### Kinesin-1 conformation can be controlled by protein design

**Fig. 2:**
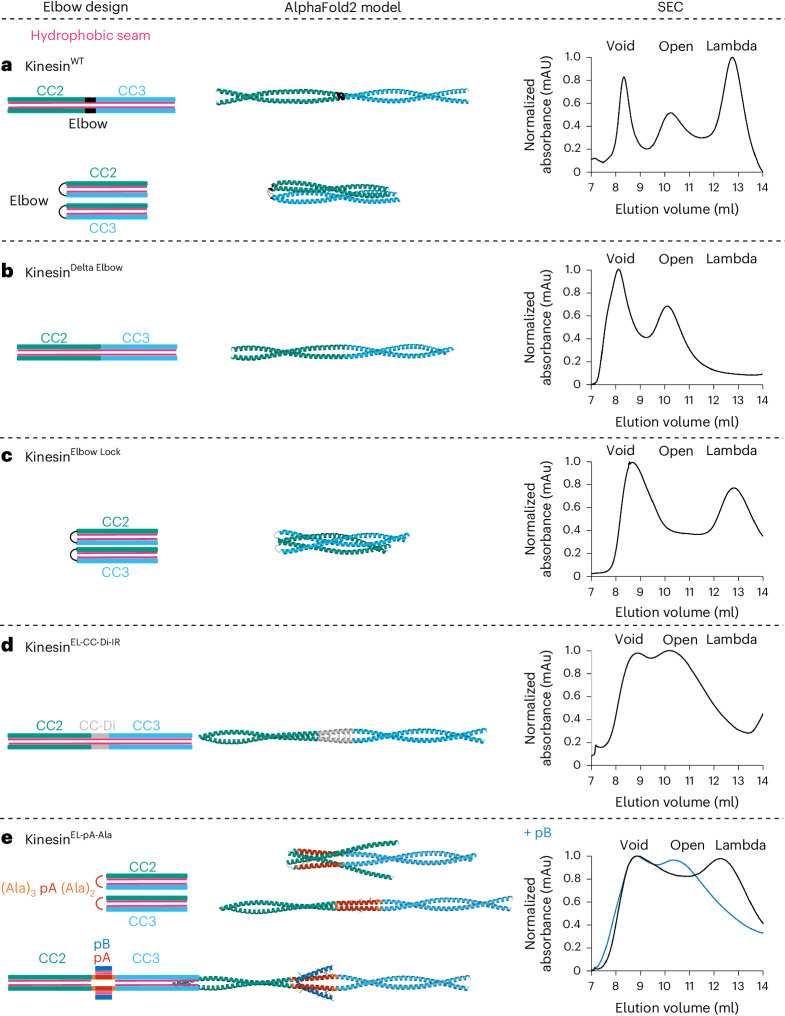
Engineering a conformational switch into kinesin-1 for allosteric activation by a de novo designed peptide. From left to right: cartoon illustrations of each elbow design; AlphaFold2 models of KHC for the CC2-linker-CC3 region; and SEC elution profiles of purified heterotetrameric KHC–KLC complexes without (black) and with (blue) peptide pB. Color scheme for the structural cartoons: KHC CC2 teal and CC3 cyan; hydrophobic seams/cores, pink; CC-Di, gray; peptide pA, red; flanks, yellow; peptide pB, blue. **a**, Wild-type kinesin-1 (Kinesin-1^WT^) exists in an equilibrium between the open and lambda states. **b**, The delta elbow variant of kinesin-1 (Kinesin-1^Delta Elbow^), with an 18-residue deletion in the elbow region, favors the open state. **c**, The elbow lock variant (Kinesin-1^Elbow Lock^), which has a five-residue deletion in the elbow region, favors the lambda state. **d**, Introduction of a 28-residue de novo designed peptide CC-Di in place of the deleted five-residue elbow of kinesin-1 (Kinesin-1^EL-CC-Di-IR^) favors the open state. **e**, Designed switchable variant Kinesin^EL-pA-Ala^ without (top) and with (bottom) peptide pB. Insertion of pA with a shifted coiled-coil register relative to CC2 and CC3 using helix-favoring (Ala)_3_ and (Ala)_2_ flanks, respectively, allows flexibility in the elbow without pB favoring the lambda particle, but induced coiled-coil formation with pB activates the switch to the open state. AlphaFold models predict both an open and alternative compact state without peptide pB, but all open models with pB. Source data

For recombinantly expressed and purified Kinesin^WT^ (composed of His-Kif5C (KHC) and KLC1 isoform A (KLC)), the lambda particle and open state (Fig. 1a,b) are apparent as two peaks after the void in size-exclusion chromatography (SEC) (Fig. 2a), and as two conformations in NS-EM^28^. We attribute the void peak to irreversible aggregation of the protein during purification and concentration. Recently, using knowledge of coiled-coil folding and assembly^30,37^, we have engineered an 18-residue elbow deletion, Kinesin^Delta Elbow^, to promote helical readthrough from CC2 to CC3 and favor the open state (Fig. 2b), which is then observed as a single peak after the void in SEC and can be visualized by NS-EM^28^. As a platform to build a switch for this new study, we sought other engineered variants that stabilize the two states of the Kinesin^WT^ complex using a combination of rational protein engineering and design, modeling with AlphaFold2 (refs. ^38,39^) and SEC as the initial experimental reporter.

First, we tested whether AlphaFold2 (refs. ^38,39^) could model KHC variants reliably. AlphaFold2 predictions of the homodimer for the wild-type CC2-elbow-CC3 region appeared to capture the two-state equilibrium. Here, the standard five AlphaFold2 models included a fully extended state in which CC2, the elbow, and CC3 form a single helix that dimerizes with an unbroken hydrophobic core, which we interpret as analogous to the open state; and four predictions of a state in which the elbow forms a loop allowing CC2 and CC3 dimers to fold back on each other (Fig. 2a and Supplementary Fig. 1). The latter closely resembles our AlphaFold2 model for the full coiled-coil architecture of the heterotetrameric lambda particle^28^. Moreover, AlphaFold2 models for the corresponding region of the Kinesin^Delta Elbow^ variant of KHC were all fully extended coiled coils (open state) consistent with our previous experiments (Fig. 2b and Supplementary Fig. 2)^28^.

To address our first objective of engineering a variant that stabilizes the folded lambda particle, we used Socket2 (ref. ^40^) to analyze the interhelical, coiled-coil, knobs-into-holes interactions predicted around the elbow of the full AlphaFold2 model for the lambda particle^28^. This indicated that a five-residue deletion from the loop, Kinesin^Elbow Lock^, might stabilize the folded-over conformation by disrupting the coiled-coil, heptad, sequence repeats and reducing conformational flexibility (Supplementary Table 1). Indeed, this modified KHC sequence returned only AlphaFold2 models compatible with the lambda particle and no extended (open) forms (Fig. 2c and Supplementary Fig. 3). Moreover, when expressed and co-purified with KLC, Kinesin^Elbow Lock^ gave a complex that eluted as a single peak after the void at the volume expected for the lambda particle, indicating that the equilibrium had indeed been completely shifted to the compact state (Fig. 2c).

Next, we asked if these key five residues in the elbow could be replaced to design a switch back to the open conformation. Our strategy was to insert a stable coiled coil into the target^18^. Specifically, we used the 28-residue, homodimeric, de novo coiled coil CC-Di^41^. We inserted this sequence into the deleted elbow region to generate Kinesin-1^EL-CC-Di-IR^, with a contiguous (in-register, IR) hydrophobic core bridging the CC2, inserted coiled-coil and CC3 domains (Fig. 2d and Supplementary Table 1). Consistent with this, AlphaFold2 models of Kinesin-1^EL-CC-Di-IR^ were solely for the extended form. Furthermore, experimentally this complex eluted after the void exclusively as the open state (Fig. 2d and Supplementary Fig. 4).

### A de novo designed peptide switches the closed to the open state

With engineered lambda and open conformations of kinesin-1 in hand, we targeted the design of a switch between these that could be triggered by a synthetic de novo designed peptide. The logic was to swap CC-Di in Kinesin-1^EL-CC-Di-IR^ for a heterodimeric coiled coil, the formation of which would drive coiled-coil extension leading to the open state and activation of the motor (Fig. 1c). We chose our heterodimeric antiparallel coiled coil, apCC-Di-AB^42^, which has two components referred to here as pA and pB (Supplementary Table 1). Individually, pA and pB are unfolded, but when combined they assemble into a stable coiled-coil heterodimer^42^. We sought to design a construct that introduced the pA sequence in place of the five-residue elbow deletion, retaining the flexibility between CC2 and CC3 required to form the lambda particle and keeping pA accessible for subsequent binding by pB.

Initially, we introduced the pA sequence in the same heptad register as CC2 and CC3 (Kinesin-1^EL-pA-IR^), analogous to Kinesin-1^EL-CC-Di-IR^ (Supplementary Table 1). Although pA is unfolded in isolation, when templated between CC2 and CC3 in this way, the sequence was predicted by AlphaFold2 to form an extended coiled coil (Supplementary Fig. 5). This was confirmed by SEC with the complex mostly in the open state (Supplementary Fig. 6a). In the context of this construct, binding of pB to pA was not modeled well by AlphaFold2 (Supplementary Figs. 6a and 7), but was confirmed experimentally by measuring the co-elution of fluorescently labeled peptide with the KHC–KLC heterotetrameric complex in SEC (Supplementary Fig. 8). Binding did not yield a conformational switch, which we attribute to the largely open population of this engineered kinesin in the absence of pB. In an attempt to address this, we introduced flexibility either side of the pA insert to prevent helical readthrough and templating by the flanking CC2 and CC3 domains. We tested triple-glycine linkers either side of pA (Kinesin-1^EL-pA-Gly^; Supplementary Table 1 and Supplementary Figs. 6b and 9). SEC confirmed a compact state for the resulting complex (Supplementary Fig. 6b). AlphaFold2 predicted that Kinesin-1^EL-pA-Gly^ could bind pB as designed, but still in a folded conformation (Supplementary Fig. 10). Binding of the peptide was confirmed by the fluorescence-based assay (Supplementary Fig. 11). However, although this second design iteration favored compact states of the kinesin complex, again it did not enable the targeted switch.

The lessons taken from these designs were that a break in coiled-coil/helical readthrough is required, but without excessive structural flexibility either side of the insert. Therefore, we placed helix-favoring alanine residues strategically either side of the pA sequence (three alanine residues preceding it and two after it) to shift its hydrophobic face by ~180˚ relative to those of the kinesin-1 CC2 and CC3 helices (Supplementary Table 1 and Fig. 2e). Thus, in this design, Kinesin-1^EL-pA-Ala^, the hydrophobic *a* and *d* sites of the *a*–*g* heptad repeats of pA should fall at solvent-exposed *f* and *b* positions of the kinesin-1 coiled-coil registers. This should not template folding of the pA insert. Without peptide pB, AlphaFold2 did not model the KHC dimer well and both compact (1/5) and open (4/5) models were returned (Supplementary Fig. 12). Encouragingly, however, exclusively open models were predicted for the pB-bound Kinesin-1^EL-pA-Ala^ dimer (Fig. 2e and Supplementary Fig. 13). Fully consistent with our design rationale, SEC revealed that the switch could be fully realized for this design (Fig. 2e and Supplementary Fig. 14): without pB, the major complex eluted after the void peak at the same volume as the lambda particle; and with pB the elution profile shifted dramatically to the open state.

### The open and closed states can be visualized in vitro

**Fig. 3:**
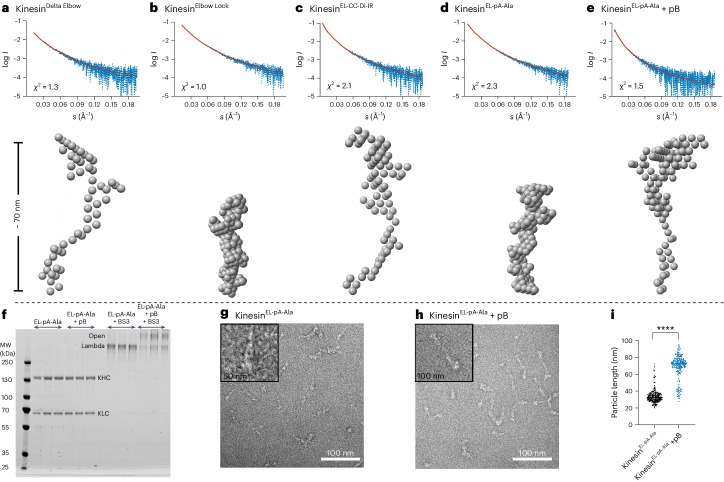
In vitro visualization of the lambda and open conformational states of kinesin-1 elbow variants. **a**–**e**, SEC–SAXS in-solution shape determination. Top: Comparison of trimmed experimental scattering profiles (blue) with theoretical scattering profiles (red) generated from ab initio models using DAMMIN. *I* is the intensity of scattering and *s* = 4πsinΘ/λ where 2Θ is the angle between the incident and scattered beam. The quality of fit is assessed using the *χ*^2^ statistic. Bottom: Representative models obtained from DAMMIN illustrating the shapes of the kinesin-1 variants. **a**, Kinesin-1^Delta Elbow^ is in the open state *D*_max_ = 74.5 nm, *χ*^2^ = 1.3. **b**, Kinesin-1^Elbow Lock^ is in the lambda state *D*_max_ = 38.3 nm, *χ*^2^ = 1.0. **c**, Kinesin-1^EL-CC-Di-IR^ is in the open state *D*_max_ = 70.0 nm, *χ*^2^ = 2.1. **d**, Kinesin-1^EL-pA-Ala^ is in the lambda state *D*_max_ = 40 nm, *χ*^2^ = 2.3. **e**, Kinesin-1^EL-pA-Ala^ + pB is in the open state *D*_max_ = 69.7 nm, *χ*^2^ = 1.5. **f**–**i**, NS-EM visualizes the peptide-induced conformational switch of kinesin-1. **f**, Coomassie-stained SDS–PAGE gel showing complexes containing Kinesin-1^EL-pA-Ala^ purified by SEC. Complexes eluted in two distinct peaks after the void for kinesin-1 without (EL-pA-Ala, peak 2) and with pB (EL-pA-Ala + pB, peak 1) sampled with 0.25 ml fractions through these peaks. Lanes marked with +BS3 show the mobility of duplicate samples after cross-linking. Cross-linked complexes formed in the presence of pB migrate slower through the gel, indicating larger (open) particles. MW, molecular weight. **g**,**h**, Representative T12 electron micrographs showing negative-stained, cross-linked samples from EL-pA-Ala (**g**) and EL-pA-Ala + pB (**h**). Scale bars, 100 nm. Zoom box size is 50 nm (**g**) and 100 nm (**h**). **i**, Quantification of particle length from panels **g** and **h** for 100 particles from each sample. Addition of pB (blue) results in an increase in the length of particles consistent with a switch from the lambda particle to the open state. Data are presented as mean ± s.e.m. and an unpaired two-tailed *t*-test was used for statistical analysis: ****P = 5.92 × 10^−101^. Source data

To examine the conformational state of kinesin-1 complexes in solution, we collected small-angle X-ray scattering (SAXS) data inline with SEC (SEC–SAXS)^43^. From the scattering profiles (Supplementary Fig. 15), we derived the radius of gyration (*R*_g_) and maximum diameter for each particle (*D*_max_) and generated bead models using DAMMIN^44^. Analysis of Kratky plots indicated flexibility in all the kinesin complexes (Supplementary Fig. 16). Notably, two different species were apparent for the Kinesin^WT^ heterotetramer, with *D*_max_ values of ~45 nm and 73 nm, respectively (Supplementary Fig. 17 and Supplementary Table 2). These are indicative of the open state and lambda particle, respectively^28^. Consistent with the predictions and SEC profiles above, Kinesin^Delta Elbow^ and Kinesin^EL-CC-Di-IR^ gave a single extended conformation of *D*_max_ ≈ 73 nm, as for the open state. In contrast, Kinesin^Elbow Lock^ and Kinesin^EL-pA-Ala^ showed single conformations of *D*_max_ ≈ 40 nm, indicating the lambda conformation (Fig. 3a–d and Supplementary Table 2). Moreover, addition of pB to Kinesin^EL-pA-Ala^ caused a shift in *D*_max_ from ~40 nm to ~70 nm (Fig. 3e and Supplementary Table 2). This provides direct biophysical evidence that binding of pB induces a conformational change in Kinesin^EL-pA-Ala^ from a closed to an open conformation.

As an orthologous approach to confirm the peptide-induced switching of Kinesin-1^EL-pA-Ala^, we trapped and visualized its states with and without pB. The protein complexes were isolated as the two major peaks from SEC and treated with the cross-linker bis(sulfosuccinimidyl)suberate (BS3)^28^. In SDS–polyacrylamide gel electrophoresis (PAGE), the complex alone gave a faster-migrating species compared to that with pB (Fig. 3f). These data indicate further that Kinesin-1^EL-pA-Ala^ alone is more compact, consistent with the lambda particle, while the complex formed with pB is more elongated. This was confirmed by NS-EM, which revealed that the major species for Kinesin-1^EL-pA-Ala^ alone resembled our previously described Kinesin-1^WT^ lambda particle^28^; namely, an ~40-nm-long V-shaped object with wide and tapered ends (Fig. 3g,i). In contrast, with pB, Kinesin-1^EL-pA-Ala^ gave predominantly long or slightly bent and thinner particles ~70–80 nm in length, similar to the open conformers of Kinesin-1^WT^ and Kinesin-1^Delta Elbow^ (Fig. 3h,i). Thus, the engineered Kinesin-1^EL-pA-Ala^ complex can access both the closed (lambda) and open states and addition of the complementary de novo designed peptide, pB, activates the switch to the latter.

### The allosteric switch can be triggered in cells

**Fig. 4:**
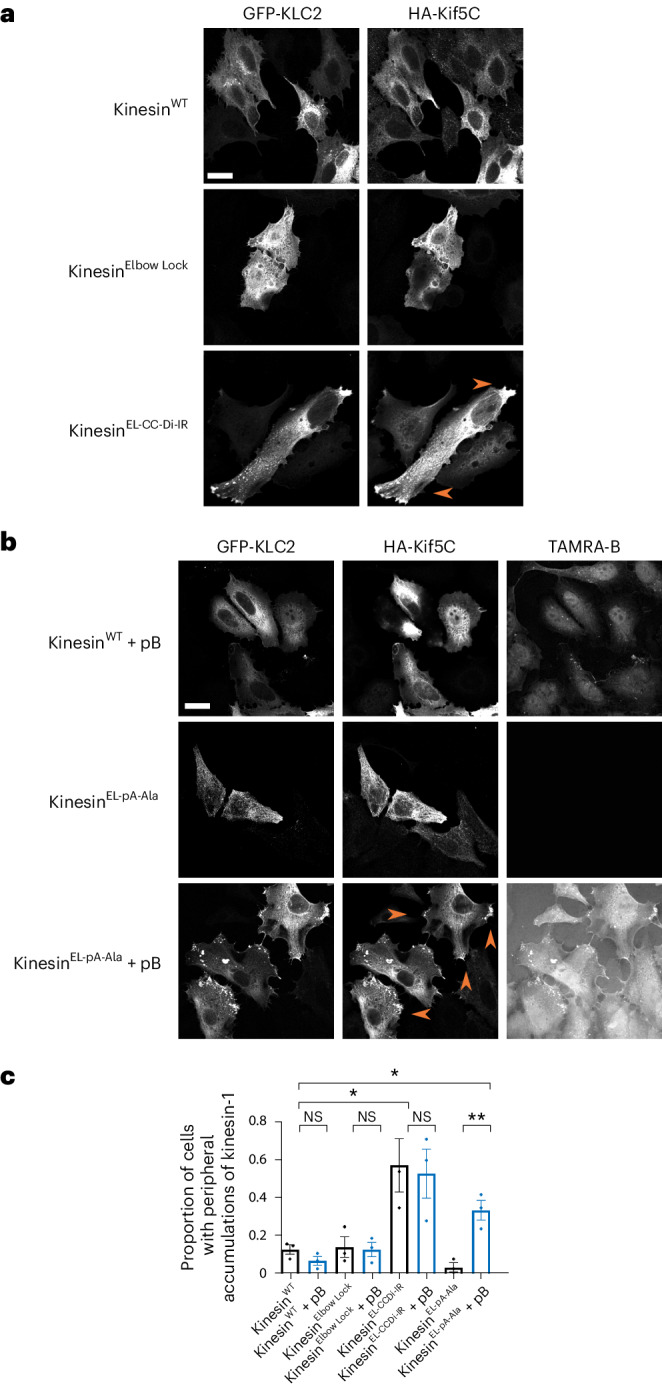
Distribution of kinesin-1 complexes in HeLa cells. **a**, HeLa cells transfected with GFP-KLC2 and HA-Kif5C (Kinesin-1^WT^, Kinesin-1^Elbow Lock^ or Kinesin-1^EL-CC-Di-IR^). Kinesin-1^WT^ and Kinesin-1^Elbow Lock^ complexes show diffuse cytosolic localization indicative of autoinhibition. Kinesin-1^EL-CC-Di-IR^ displays extended cell morphology and peripheral accumulations of kinesin-1 at cell vertices. Scale bar, 20 µm. **b**, HeLa cells transfected with GFP-KLC2 and HA-Kif5C (Kinesin-1^WT^, Kinesin-1^EL-pA-Ala^) with/without treatment with 2 μM TAMRA-labeled pB for 1 h. pB is cell penetrating as shown by TAMRA fluorescence in the cytoplasm and nucleus of Kinesin-1^WT^ transfected cells. pB binds to and colocalizes with Kinesin-1^EL-pA-Ala^ complexes in peripheral accumulations at cell vertices. Scale bar, 20 µm. **c**, Quantification of proportion of cells with peripheral accumulations of kinesin-1 in panels **a** and **b** in a minimum of 45 cells (102, 84, 45, 48, 62, 52, 103, 70 cells from left to right) pooled from three independent experiments. Additional controls of Kinesin-1^Elbow Lock^ and Kinesin-1^EL-CC-Di-IR^ with pB show no change on addition of peptide. Data are presented as mean ± s.e.m. and an unpaired two-tailed *t*-test was used for statistical analysis. NS, not significant; **, Kinesin-1^EL-pA-Ala^ versus Kinesin-1^EL-pA-Ala+pB^ *P* = 0.0064; *, Kinesin-1^WT^ versus Kinesin-1^EL-pA-Ala+pB^ *P* = 0.0237; Kinesin-1^WT^ versus Kinesin-1 ^EL-CC-Di-IR^ *P* = 0.0353. Source data

Finally, we tested whether the peptide-induced conformational switch could allosterically activate kinesin-1 activity in cells. HeLa cells were transiently transfected with two genes to express kinesin-1 heterotetramers. For visualization, KLC was conjugated to a green fluorescent protein (GFP-KLC2) and KHC to a hemagglutinin tag (HA-Kif5C). The KHC gene was mutated to incorporate the elbow variants and then imaged to examine kinesin-1 localization by fluorescence light microscopy. Cells expressing Kinesin-1^WT^ and Kinesin-1^Elbow Lock^, which favor the lambda particle conformation, displayed diffuse cytosolic localization of both KHC and KLC, characteristic of autoinhibited kinesin. By contrast, Kinesin-1^EL-CC-Di-IR^, which favors the open state, gave a different phenotype of extended cells with peripheral accumulations of kinesin-1 (Fig. 4a and Supplementary Fig. 18). This indicates that this variant is a constitutively active kinesin motor that moves along microtubules to the cell periphery. pB is also a cell-penetrating peptide that enters directly into the cytoplasm and nuclei of HeLa cells^42^. In cells expressing Kinesin-1^EL-pA-Ala^, adding fluorescently labeled pB to the media resulted in its colocalization with KLCs and KHCs and caused a dramatic redistribution of kinesin-1 complexes to peripheral accumulations (Fig. 4b). In control experiments without pB, Kinesin-1^EL-pA-Ala^ resembled the diffuse distribution of Kinesin-1^WT^ and Kinesin-1^Elbow Lock^. Together, these experiments show that cell-penetrating pB can target the engineered Kinesin-1^EL-pA-Ala^ to effect inducible allosteric activation in living cells (Fig. 4b,c).

## Discussion

In summary, we have applied rational peptide and protein design principles to engineer a peptide-induced conformational switch within a molecular motor that can be triggered in vitro and to allosterically activate kinesin-1 in cells using an exogenous reagent. In doing so, we show how a fundamental understanding of sequence-to-structure relationships of coiled-coil domains can enable the design of reagents to target them in complex biological machines.

This design-led approach also provides important insight on the mechanism of kinesin-1 autoregulation and, going forward, our ability to target it. The capacity to stabilize or to relieve autoinhibition via subtle manipulations of the elbow region indicates that this hinge is a key and finely tuned regulatory switch of the kinesin-1 motor complex. Indeed, introduction of a de novo designed coiled-coil peptide with nM affinity^42^ is sufficient to switch the conformation from the folded lambda particle to an open state and promote activity. From this, we posit that the sum of all other interactions involved in autoinhibition must be less than the free energy change associated with the designed binding event. Therefore, it seems that the primary function of the motor–IAK autoinhibitory interactions is not to drive closure to the lambda state, but rather to supress enzymatic activity and modulate cargo-binding properties within this state. This notion is consistent with recent observations that mutation of the IAK motif is not sufficient to open and activate the complex^29,45^.

In nature, kinesin-1 motors are activated through binding of a diverse array of cargo via adaptor proteins^26,46^, but the underlying mechanisms remain unclear. A key inference from our study is that the primary function of many coiled coil-binding cargo-adaptor proteins and microtubule-associated proteins (such as MAP7) is to perturb the finely balanced equilibrium between the lambda and open state^26,47,48^. Our findings indicate that the autoinhibitory mechanism based around this coiled-coil switch is poised to respond to both endogenous and exogenous inputs. Therefore, it may be amenable to designing new reagents that target the endogenous protein to boost or supress kinesin-1-mediated intracellular transport where it is implicated in disease.

In addition, the constructs that we have derived provide valuable tools for future studies into both the structure and function of kinesin-1 by stabilizing either the autoinhibited or active state without removing key cargo recognition and regulatory components of the protein complex. Our system offers an inducible means for in-cell activation of kinesin-1 without perturbing key cargo binding and enzymatic activities.

A long-standing question is the extent to which the kinesin-1 complex opens to engage in cargo transport. Although most models assume that the complex fully extends, others suggest that a more compact form is responsible for cargo transport^49,50^. Our finding that inducible peptide-driven extension is sufficient to activate the complex is consistent with the extension model. Our ability to make and test model-based predictions for successful protein design indicates that the elbow sequence is highly evolved to adopt two distinct states—a flexible hinge and a rigid readthrough of helical, heptad repeats from CC2 to CC3. Our findings also suggest limited flexibility in the rest of the coiled-coil scaffold. We propose that rigid readthrough (modulated by cargo-adaptor binding) could represent the functional active complex, and should be a focus for future experiments.

The kinesin-1 motor system offered an attractive target to test our engineering and design approach to effect an allosteric switch in a natural enzyme. We believe that this approach may be developed in various ways. First, the principles that we have devised should be applicable to other proteins whose conformational state is regulated by hinging coiled coils that can switch between loop and folded conformations^18,51–55^. Second, reagents of the type we present could be developed to target natural coiled-coil domains more generally. This work demonstrates the importance of balancing interaction affinities within protein complexes and with their effectors. Potentially, our approach ushers in a new era of quantitative chemical biology. If realized, this would open the door to larger scale efforts to target these domains, which comprise up to 15% of the human proteome^56,57^.

## Methods

### AlphaFold2 predictions

Predictions were made using the Google Colab AlphaFold Notebook with a Colab Pro+ subscription^38,39^. The algorithm was asked to model a 1:1 homodimer composed of rat KIF5C (NP_001101200.1) residues I590 to L772 or a 2:2 heterotetramer of these sequences with peptide pB. These boundaries were established by combining insight from Marcoil^58,59^ predictions and Socket2 (ref. ^40^) analysis of a larger model described previously^28^. Default parameters were used, and the amber relaxation step was enabled. The five output models were downloaded in pdb format, aligned and prepared for presentation using PyMOL. Model statistics are as presented by the software.

### Plasmids used in this study

Previously, rat KIF5C (corresponding to residues 2 to 955), was amplified by PCR from an HA-tagged expression construct and ligated into the His-3C cleavage pMW bacterial expression vector, and mouse KLC1 was amplified by PCR and ligated into pET28a using NdeI/XhoI sites with the N-terminal His-thrombin cleavage tag removed by site-directed mutagenesis^28,60^. Clonal genes containing fragments of the elbow designs described in this study were synthesized by Twist Bioscience and cloned into pMW-His-rat *Kif5C* using BrsGI/EcoR1 sites. These constructs were subcloned into a CB6 mammalian expression vector using NotI/EcoRI sites.

### Protein expression and purification

Heterotetrametric kinesin-1 was expressed in BL21(DE3) cells using a two-plasmid system. BL21(DE3) cells, transformed with both KIF5C and KLC1 expression plasmids, were used to inoculate 1 liter of LB cultures supplemented with both ampicillin and kanamycin. Cells were grown with shaking at 37 °C until optical density reached 0.8, before the cultures were cooled to 18 °C and protein expression was induced with 0.3 μM isopropyl-β-d-thiogalactopyranoside. Following shaking incubation at 18 °C overnight, cells were collected by centrifugation at 6,000*g* at 4 °C for 15 min and resuspended (10 ml per 1 liter of original culture) in 20 mM HEPES (pH 7.4), 300 mM NaCl and 40 mM imidazole before being stored at −20 °C. Frozen pellets were thawed and diluted in 25 ml of buffer consisting of 40 mM HEPES (pH 7.4), 500 mM NaCl, 40 mM imidazole, 5% (v/v) glycerol and 5 mM β-mercaptoethanol with a Roche complete protease inhibitor tablet added. Bacteria were lysed by sonication, 0.5 s on and 10 s off, at 70% amplitude for 7 min and 30 s in an ice bath. Lysate was clarified by centrifugation at 35,000*g* on a JA-20 rotor for 40 min at 4 °C. Clarified lysate was filtered (0.45 μm) before being loaded onto a His-Trap (Sigma-Aldrich) column. The column was washed in buffer and eluted with a gradient of 40 to 500 mM imidazole. Eluted protein was concentrated by ultrafiltration in a 10,000-Da molecular weight cutoff filter (Cytiva), before snap-freezing in liquid nitrogen.

### Size-exclusion chromatography

Proteins were thawed and incubated for 1 h at 4 °C with agitation in the presence of peptide pB or a vehicle control (1 µl H_2_O). Proteins were further purified by SEC using a Superose6 10/300 column (Cytiva), in 20 mM HEPES (pH 7.4), 150 mM NaCl, 1 mM MgCl_2_, 0.1 mM adenosine 5′-diphosphate and 0.5 mM tris(2-carboxyethyl)phosphine (Sigma-Aldrich) at a flow rate of 0.5 ml min^−1^. Elution of protein was monitored by following absorbance at 280 nm and individual fractions were run on a Coomassie-stained gel to confirm size and purity. Elution of peptide pB was monitored by measuring fluorescence at 555 nm of individual fractions in a plate reader. Measurements were repeated a minimum of three times and one representative trace, plotted in Prism 9, is shown for clarity.

### Peptide synthesis

As described previously^42^, peptide pB was prepared by standard Fmoc solid-phase peptide synthesis on a 0.1 mM scale using CEM Liberty Blue automated peptide synthesis apparatus with inline UV monitoring. Activation was achieved with DIC/Oxyma. Fmoc deprotection was performed with 20% v/v morpholine/dimethyl formamide (DMF). Double couplings were used for β-branched residues and the subsequent amino acid. Synthesis was from C to N terminus as the C-terminal amide on Rink amide resin and labeling achieved by addition of TAMRA (0.1 mM, 2 equiv.), HATU (0.095 mM, 1.9 equiv.) and DIPEA (0.225 mM, 4.5 equiv.) in DMF (3 ml) to DMF washed peptide resin (0.05 mM) with agitation for 3 hours. Resin was washed with 20% piperidine in DMF (5 ml) for 2 × 30 minutes to remove any excess dye. All manipulations were carried out under foil to exclude light. Peptide pB was cleaved from the solid support by addition of TFA (9.5 ml), TIPS (0.25 ml) and water (0.25 ml) for 3 hours with shaking at room temperature. The cleavage solution was reduced to approximately 1 ml under a flow of nitrogen. Crude peptide was precipitated upon addition of ice-cold diethyl ether (40 ml) and recovered via centrifugation. The resulting precipitant was dissolved in 1:1 acetonitrile and water (~15 ml) and lyophilized to yield crude peptide as a solid.

### Peptide purification

As described previously^42^, peptide pB was purified by reverse-phase HPLC on a Phenomenex Luna C18 stationary phase column (150 × 10 mm, 5 μM particle size, 100 Å pore size) using a preparative JASCO HPLC system. A linear gradient of 20–80% acetonitrile and water (with 0.1% TFA) was applied over 30 minutes. Chromatograms were monitored at wavelengths of 220 and 280 nm. The peptide was confirmed using MALDI-TOF mass spectrometry using a Bruker ultrafleXtreme II instrument in reflector mode. Peptide was spotted on a ground-steel target plate using α-cyano-4-hydroxycinnamic acid (CHCA) as the matrix. Masses were measured to 0.1% accuracy. Peptide purity was determined using a JASCO analytical HPLC system, fitted with a reverse-phase Kinetex C18 analytical column (100 × 4.6 mm, 5 µm particle size, 100 Å pore size)^42^. Fractions containing pure peptide were pooled and lyophilized. Peptide was dissolved in buffer and concentration determined by UV–visible at 280 nm on a ThermoScientific Nanodrop 2000 spectrophotometer by measurement of the UV absorbance at 555 nm (absorbance *ε*_555_(TAMRA) = 85,000 mol^−1^ cm^−1^).

### Small-angle X-ray scattering

SAXS data were collected at Diamond Light Source Synchrotron (Didcot, UK) on the B21 beamline, with an HPLC system upstream (Supplementary Table 3)^43^. As before, a Superose6 10/300 column (Cytiva) was equilibrated in 20 mM HEPES (pH 7.4), 150 mM NaCl, 1 mM MgCl_2_, 0.1 mM adenosine 5′-diphosphate and 0.5 mM tris(2-carboxyethyl)phosphine (Sigma-Aldrich). Purified protein (95 ml of Kinesin-1^WT^ 73 µM, Kinesin-1^Delta Elbow^ 31 µM, Kinesin-1^Elbow Lock^ 41 µM, Kinesin-1^EL-CC-Di-IR^ 23 µM or Kinesin-1^EL-pA-Ala^ 26 µM or Kinesin-1^EL-pA-Ala + pB^ 33 µM)) was injected onto the pre-equilibrated column. The flow rate of the column was maintained at 0.5 ml min^−1^ with eluted samples exposed to X-rays for a 6-second exposure time. Data were analyzed in CHROMIXS^61^. The region of an individual peak corresponding to a single conformation was selected based on radius of gyration (*R*_g_) residuals and subtracted from the selected buffer region, scaled and averaged to obtain a Guinier region. Further data processing was performed in PRIMUSQT^62^. The pair distribution function (*P*(*r*)) was calculated by GNOM^63^. The data were truncated at low angle for Kinesin-1^WT^ (open and lambda) (10 points), Kinesin-1^Delta Elbow^ (50 points), Kinesin-1^Elbow Lock^ (50 points) and Kinesin-1^EL-pA-Ala^ (10 points) due to higher error at low scattering angles and to remove scattering from higher oligomers despite the attempted separation. The *D*_max_ and *R*_g_ values obtained from *P*(*r*) were calculated for all constructs and the output file was used for the ab initio modeling in a slow mode using DAMMIN^44^.

### Negative-stain electron microscopy

For NS-EM, freshly eluted proteins from size exclusion were cross-linked with 0.6 mM BS3 (Thermo Fisher Scientific) for 30 min at room temperature. Cross-linked proteins were diluted to 0.003 mg ml^−1^ in size-exclusion buffer, and 5 μl was pipetted onto a freshly glow-discharged grid (300-mesh copper with formvar/carbon support, TAAB) and incubated at room temperature for 1 min. Grids were manually blotted, washed in 3% uranyl acetate (UA) and stained in 20 μl of 3% UA for 20 seconds, followed by a final wash in 3% UA, with the excess blotted away. The samples were air dried. Micrographs of grids were acquired on an FEI 120-kV BioTwin equipped with an FEI Ceta 4*k* × 4*k* charge-coupled device camera at ×49,000 magnification, corresponding to a pixel size of 2.04 Å per pixel. Particle length was measured using ImageJ from several micrographs for each complex and plotted in Prism v.9.

### Cell culture

HeLa cells were maintained in high glucose Dulbecco’s modified Eagle’s medium (Gibco Invitrogen) with 10% (v/v) fetal calf serum (Sigma-Aldrich) and 5% penicillin/streptomycin (PAA) (herein referred to as DMEM) at 37 °C and 5% CO_2_. For transfection, cells were seeded in six-well plates on fibronectin-coated 13-mm coverslips at a density of 1 × 10^5^ cells per well and incubated at 37 °C and 5% CO_2_ for 16 h before transfection. Cells were transfected with 0.4 µg DNA using Effectene transfection reagent according to the manufacturer’s instructions (Qiagen). After transfection cells were incubated at 37 °C, 5% CO_2_ for 16 h. Peptide treatments were in 1 ml DMEM, 37 °C and 5% for 1 h with 2 µM TAMRA-pB or vehicle control (1 µl H_2_O). Cells were fixed by addition of 4% paraformaldehyde in PBS at room temperature for 10 minutes (2 ml per well for six-well plate) and washed 3× with PBS before staining with a mouse monoclonal anti-HA antibody (1:1,000, Sigma-Aldrich, catalog no. H3663) and goat anti-mouse IgG secondary antibody, Alexa Fluor 647 (1:400, Thermo Fisher). Confocal images were collected using a Leica SP5II system with a ×63 objective running Leica LAS X and are presented as maximum intensity projections. Figures were assembled using ImageJ in conjunction with Inkscape. Image analysis was conducted in ImageJ and data plotted in Prism 9.

### Reporting summary

Further information on research design is available in the Nature Portfolio Reporting Summary linked to this article.

## Online content

Any methods, additional references, Nature Portfolio reporting summaries, source data, extended data, supplementary information, acknowledgements, peer review information; details of author contributions and competing interests; and statements of data and code availability are available at 10.1038/s41589-024-01640-2.

### Supplementary information

Supplementary Information Supplementary Tables 1–3 and Figs. 1–18.

Reporting Summary

### Source data

Source Data Fig. 2 Numerical source data for size-exclusion chromatography.

Source Data Fig. 3 Numerical source data for SAXS and NS-EM.

Source Data Fig. 4 Numerical source data for cell phenotypes.

## Data Availability

All data needed to interpret, verify and extend the study presented in this article are available in the manuscript, supplementary information and source files. In addition, the raw data used in this publication have been deposited in the Zenodo repository at 10.5281/zenodo.11061566 (ref. ^64^). Source data are provided with this paper.
