# Supplementary material for: A de novo designed coiled coil-based switch regulates the microtubule motor kinesin-1
Source: Nat Chem Biol. 2024 Jun 7;20(7):916–23. doi: 10.1038/s41589-024-01640-2 (PMC11213707; doi:10.1038/s41589-024-01640-2)
Supplement: Supplementary file 1 — Supplementary Tables 1–3 and Figs. 1–18. [file 41589_2024_1640_MOESM1_ESM.pdf]

nature chemical biology

Supplementary information https://doi.org/10.1038/s41589-024-01640-2

A de novo designed coiled coil-based switch regulates the microtubule motor kinesin-1

In the format provided by the authors and unedited

Table of contents

Supplementary Tables S1 to S3 Pages 3-4

Supplementary Figures S1 to S18 Pages 5-20

Table S1: Coiled coil register assignment of elbow designs used in this study		
**Kif5C CC2 674→**<br>***abcdefg abcd***<br>EKMHEVS FQDK	**Elbow**	**CC3 → 698**<br>***cd abcdefg***<br>TR LQDAEEV
Kinesin^{WT}	EKEHL	
Kinesin$^{Elbow\ Lock}$	-	
Register$^{CC2/CC3/AB}$	***ef gabcdef gabcdef gabcdef gab***	
Kinesin$^{EL-CC-Di-IR}$	KQ EIAAIKK ENAALKW EIAALKQ EIA	
Kinesin$^{EL-A-IR}$	EQ ELAALDQ EIAAAEQ ELAALDW QIQ	
Kinesin$^{EL-A-Gly}$	*GGG* QLEQ ELAALDQ EIAAAEQ ELAALDW QIQ *GGG*	
Register$^{CC2/CC3}$	***efgabcd efgabcd efgabcd efgabcd efgab***	
Kinesin$^{EL-A-Ala}$<br>Register^{AB}	*AAA*QLEQ ELAALDQ EIAAAEQ ELAALDW QIQ*AA*<br>***cdef gabcdef gabcdef gabcdef gab***	
Peptide-B	*G* QLKQ RRAALKQ RIAALKQ RRAALKW QIQ *G*	

Table S2. Size distribution parameters D_{max} and R_g values obtained from pair distance distribution ($P(r)$) analysis

Elbow Design	D_{max} (nm)	R_g (nm)
Kinesin$^{WT: Lamda}$	45.0	13.8
Kinesin$^{WT: Open}$	72.9	20.8
Kinesin$^{Delta\ Elbow}$	74.5	22.2
Kinesin$^{Elbow\ Lock}$	38.3	10.6
Kinesin$^{EL-CC-Di-IR}$	70.0	21.5
Kinesin$^{EL-A-Ala}$	40.0	13.1
Kinesin$^{EL-A-Ala\ +\ Peptide\ B}$	69.7	20.9

**Table S3. SEC-SAXS Data collection parameters**	
**Source Type**	Bending magnet
**Beamline**	B21, biological X-ray scattering
**Detector**	Eiger 4M (Dectris)
**Beam size at the focal point at detector (FWHM)**	34 x 40 µm (horizontal vertical)
**Beam size at sample**	01102 x 240 µm
**Wavelength range**	0.89 – 1.3 Å
**Mode/Column**	SEC/Superose 6 (10/300)
**Exposure time**	3 s

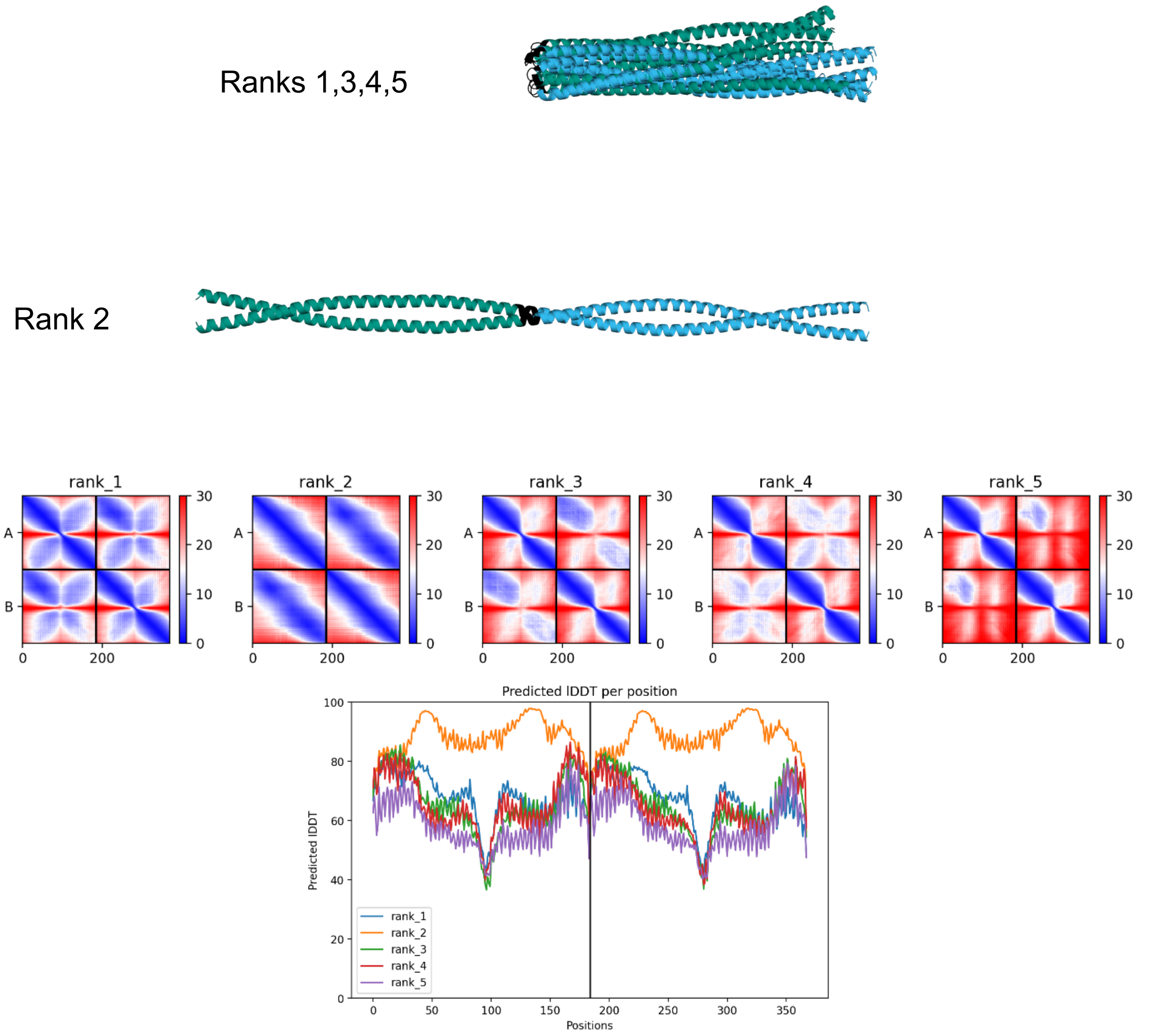

Sequence:
iskmksevkslvnrskqlesaqtdsnrkmnaserelaacqllisqheakiksltdymqnmeqkrrqle
esqdslseelaklraqekmhevsfqdkekehltrlqdaeevkkaleqqmeshreahqkqlsrlrdeie
ekqriideirdlnqklqleqerlssdynklkiedqerevklekllllln

Fig. S1. Alignment of AlphaFold2 models of Kinesin-1^{WT} CC2-elbow-CC3 with corresponding PAE and pLDDT plots and sequence. 4 aligned models (ranks 1,3,4,5) of Kinesin-1^{WT} CC2-elbow-CC3 have a folded structural prediction with a flexible elbow (black) between CC2 (teal) and CC3 (cyan) The remaining model predicts an extended coiled coil conformation (rank 2).

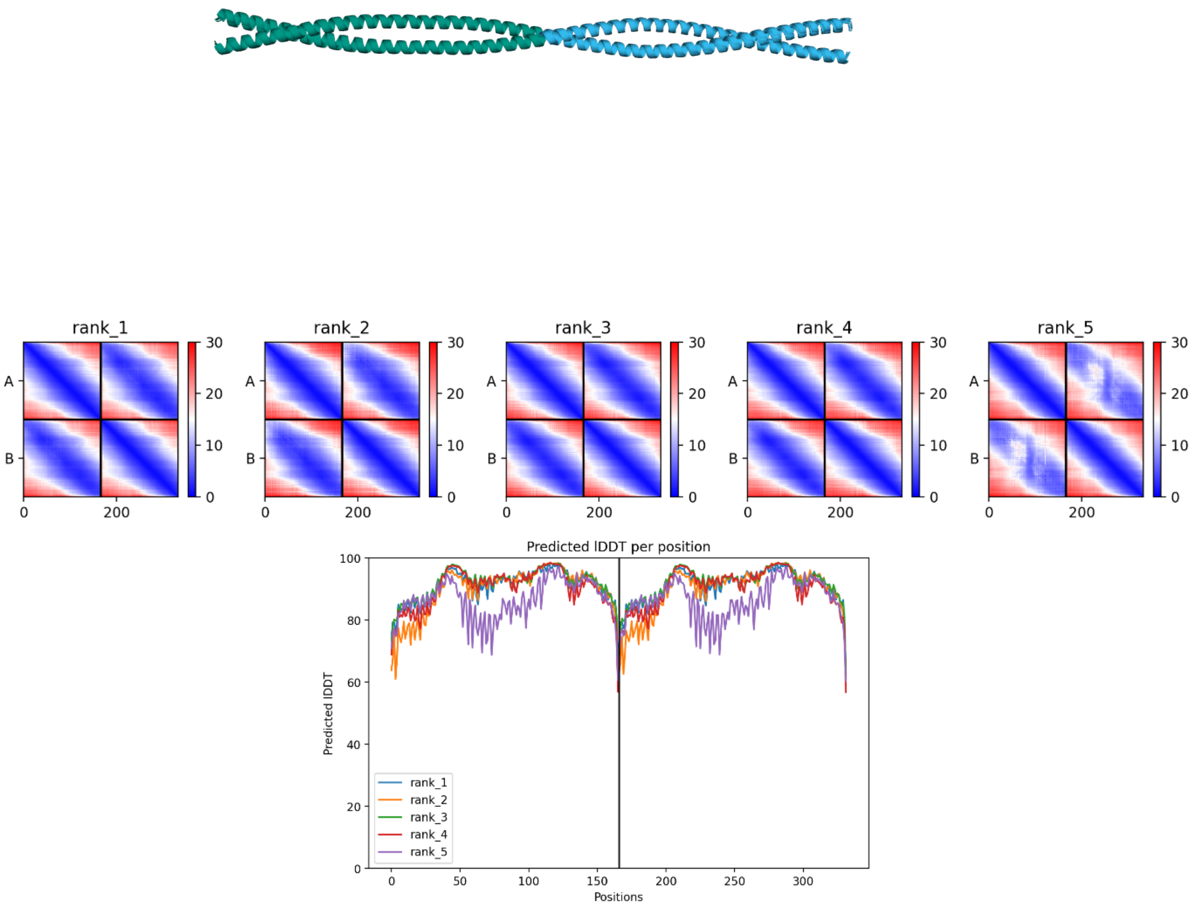

Sequence:
iskmksevkslvnrskqlesaqtdsnrkmnaserelaacqllisqheakiksltdymqnmeqkrrqle
esqdslseelaklraqlqdaeevkkaleqqmeshreahqkqlsrlrdeieekqriideirdlnqklql
eqerlssdynklkiedqerevkleklllln

Fig. S2. Alignment of AlphaFold2 models of Kinesin-1$^{\text{Delta Elbow}}$ CC2-elbow-CC3 with corresponding PAE and pLDDT plots and sequence. All five models closely overlay with the same extended coiled coil structural prediction, CC2 (teal) and CC3 (cyan).

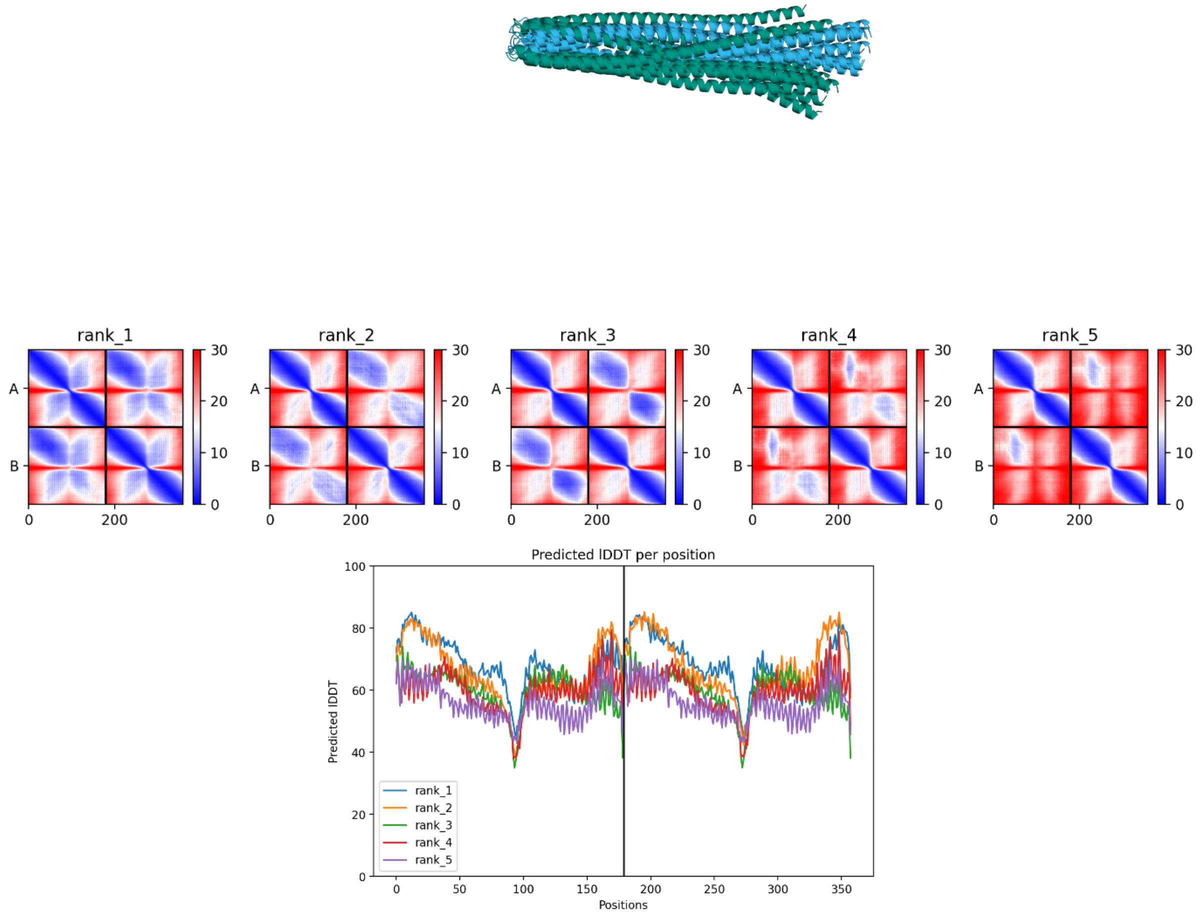

Sequence:
iskmksevkslvnrskqlesaqtdsnrkmnaserelaacqllisqheakiksltdymqnmeqkrrqle
esqdslseelaklraqekmhevsfqdktrlqdaeevkkaleqqmeshreahqkqlsrlrdeieekqri
ideirdlnqklqleqerlssdynklkiedqerevkleklllln

Fig. S3. Alignment of AlphaFold2 models of Kinesin-1$^{Elbow\ Lock}$ CC2-elbow-CC3 with corresponding PAE and pLDDT plots and sequence. All five models have a folded structural prediction with a flexible hinge between CC2 (teal) and CC3 (cyan).

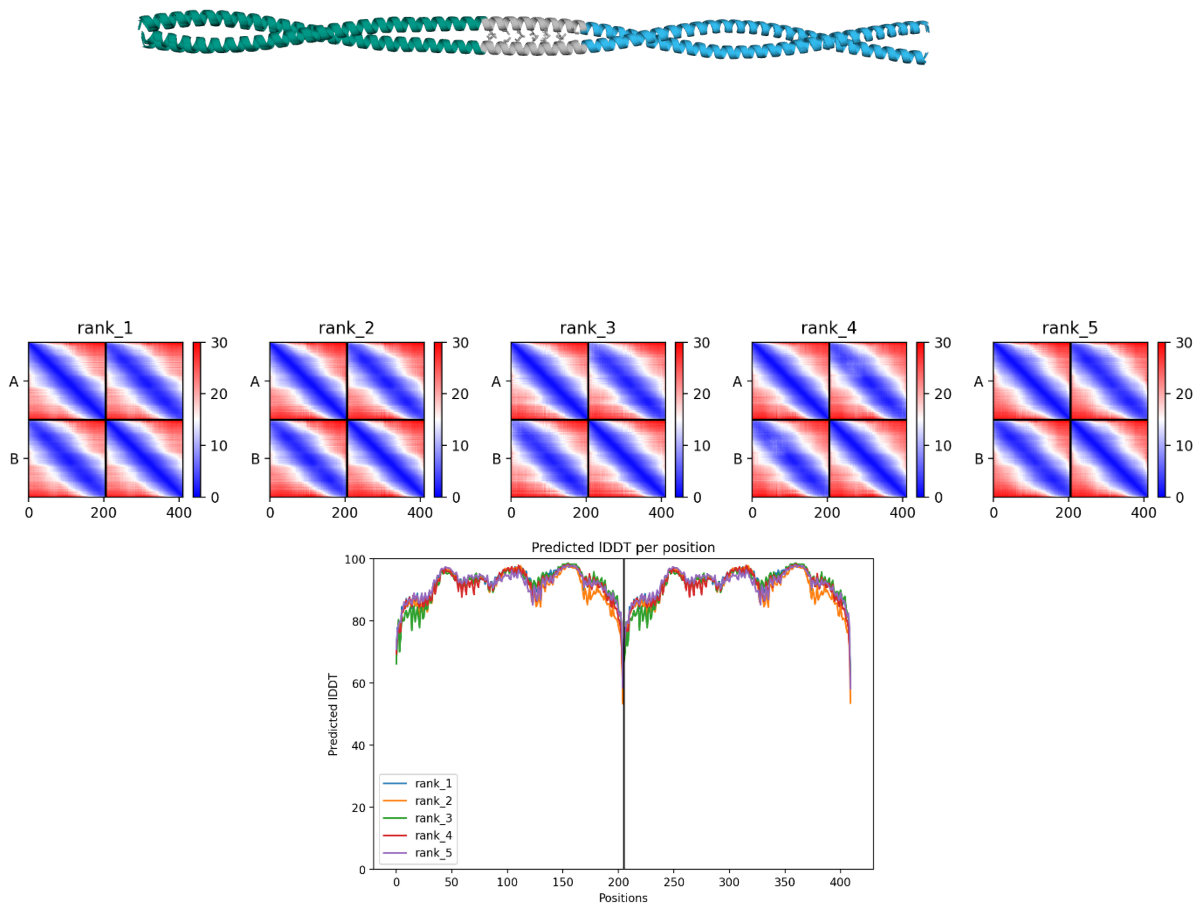

Sequence:
iskmksevkslvnrskqlesaqtdsnrkmnaserelaacqllisqheakiksltdymqnmeqkrrqle
esqdslseelaklraqekmhevsfqdkKQEIAAIKKENAALKWEIAALKQEIAtrlqdaeevkkaleq
qmeshreahqkqlsrlrdeieekqriideirdlnqklqleqerlssdynklkiedqerevklekllll
n

Fig. S4. Alignment of AlphaFold2 models of Kinesin-1$^{\text{EL-CC-Di-IR}}$ CC2-elbow-CC3 with corresponding PAE and pLDDT plots and sequence. All five models closely overlay with the same extended coiled coil structural prediction, CC2 (teal) and CC3 (cyan) with CC-Di in grey. Side chains are shown as sticks for the hydrophobic core of CC-Di.

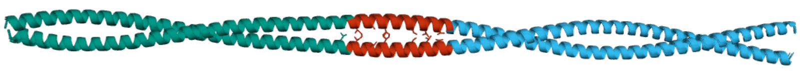

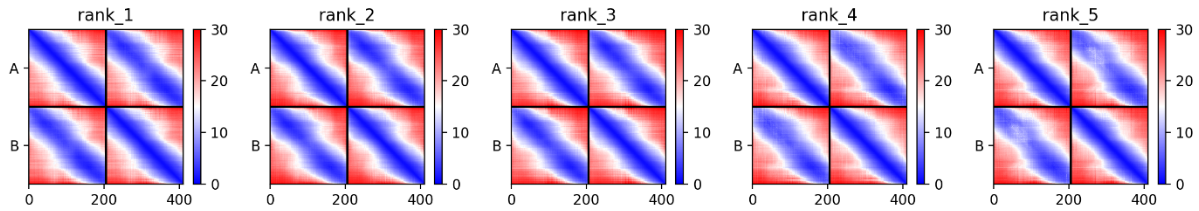

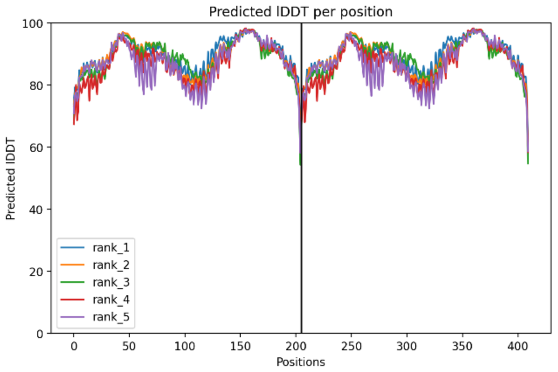

Sequence:

iskmksevkslvnrskqlesaqtdsnrkmnaserelaacqllisqheakiksltdymqnmeqkrrqle
esqdslseelaklraqekmhevsfqdkEQELAALDQEIAAAEQELAALDWQIQtrlqdaeevkkaleq
qmeshreahqkqlsrlrdeieekqriideirdlnqklqleqerlssdynklkiedqerevklekllll
n

Fig. S5. Alignment of AlphaFold2 models of Kinesin-1$^{EL\text{-}pA\text{-}IR}$ CC2-elbow-CC3 with corresponding PAE and pLDDT plots and sequence. All five models closely overlay with the same extended coiled coil structural prediction, CC2 (teal) and CC3 (cyan) with pA in red. Side chains are shown as sticks for the hydrophobic core of pA.

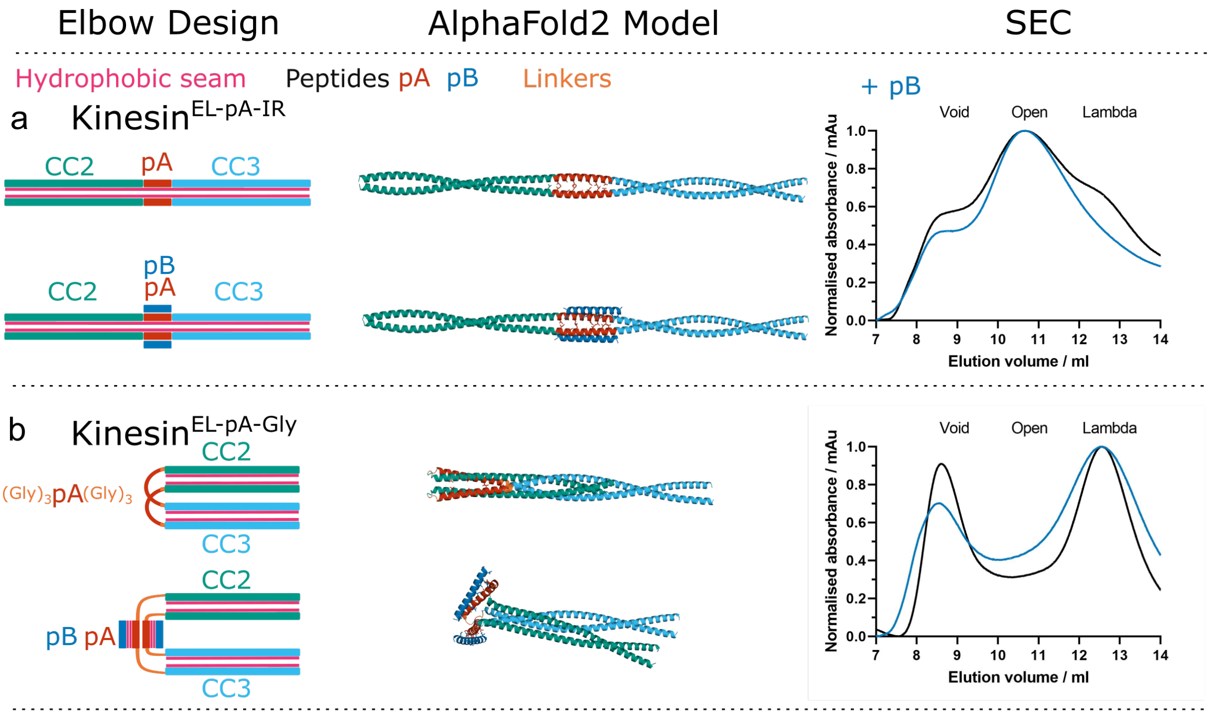

Fig. S6. Designing an allosteric switch into kinesin-1 that is activated by a *de novo* designed peptide. From left to right: cartoon illustrations of each elbow design without (top) and with (bottom) peptide pB; AlphaFold2 models of KHC for the CC2-linker-CC3 region without (top) and with (bottom) peptide pB; and size-exclusion chromatography (SEC) elution profiles of purified heterotetrameric KHC-KLC complexes without (black) and with (blue) peptide pB. Colour scheme for the structural cartoons: KHC CC2 teal, CC3 cyan; hydrophobic seams/cores, magenta; peptide pA, red; linkers, orange; and peptide pB, blue. **a.** Insertion of pA in register (IR) between CC2 and CC3 drives helical read through and favours the open state without or with pB. **b.** Insertion of pA between flexible $(Gly)_3$ linkers after CC2 and before CC3 favours the lambda particle without or with pB.

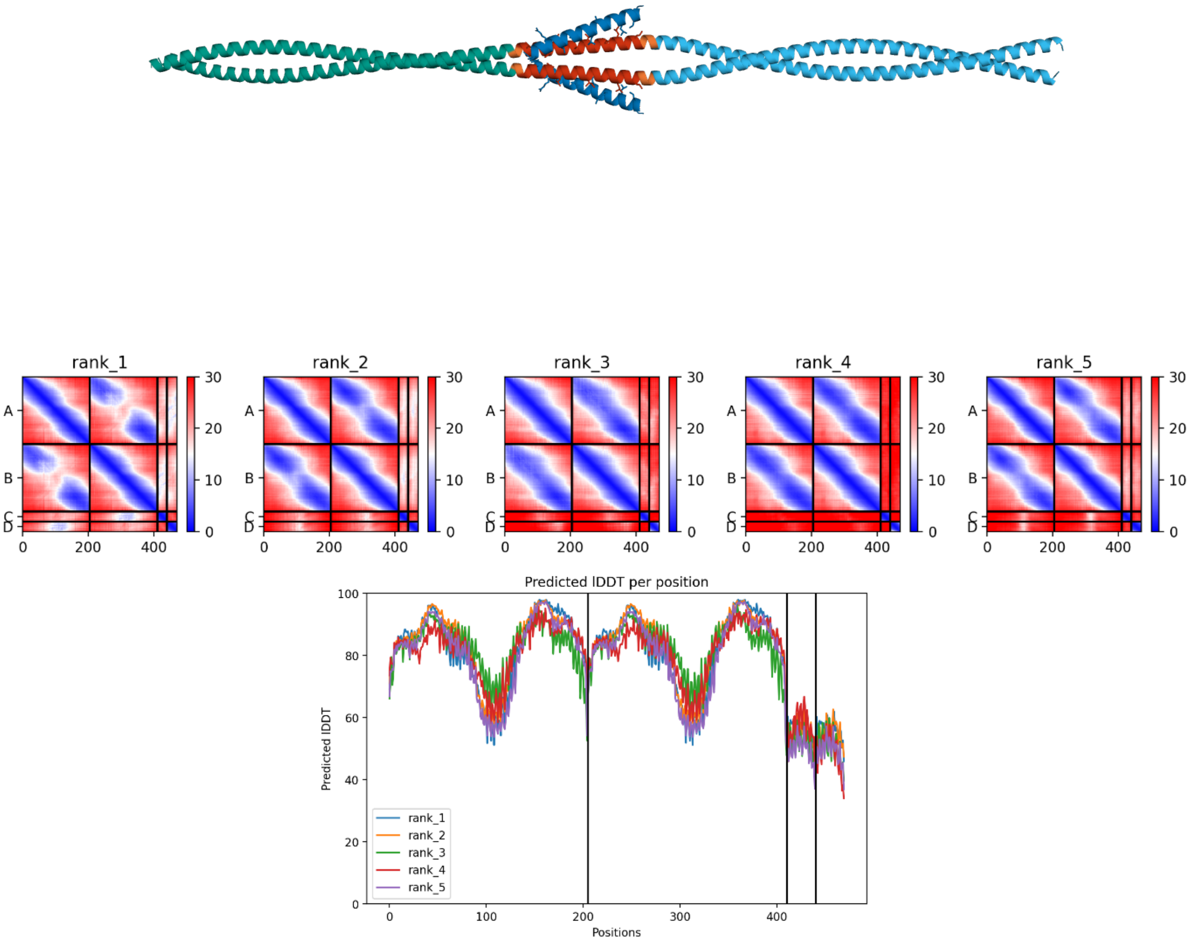

Sequence:

iskmksevkslvnrskqlesaqtdsnrkmnaserelaacqllisqheakiksltdymqnmeqkrrqle
esqdslseelaklraqekmhevsfqdkEQELAALDQEIAAAEQELAALDWQIQtrlqdaeevkkaleq
qmeshreahqkqlsrlrdeieekqriideirdlnqklqleqerlssdynklkiedqerevklekllll
n

Peptide pB: GQLKQRRAALKQRIAALKQRRAALKWQIQG

Fig. S7. Alignment of AlphaFold2 models of Kinesin-1$^{EL\text{-}pA\text{-}IR}$ CC2-elbow-CC3 with peptide pB with corresponding PAE and pLDDT plots and sequences. All five models of Kinesin-1$^{EL\text{-}pA\text{-}IR}$ CC2-elbow-CC3 closely overlay with the same extended coiled coil structural prediction, CC2 (teal) and CC3 (cyan) with pA in red. The placement of peptide pB (blue) is more varied. Side chains are shown as sticks for the hydrophobic core of pA and pB.

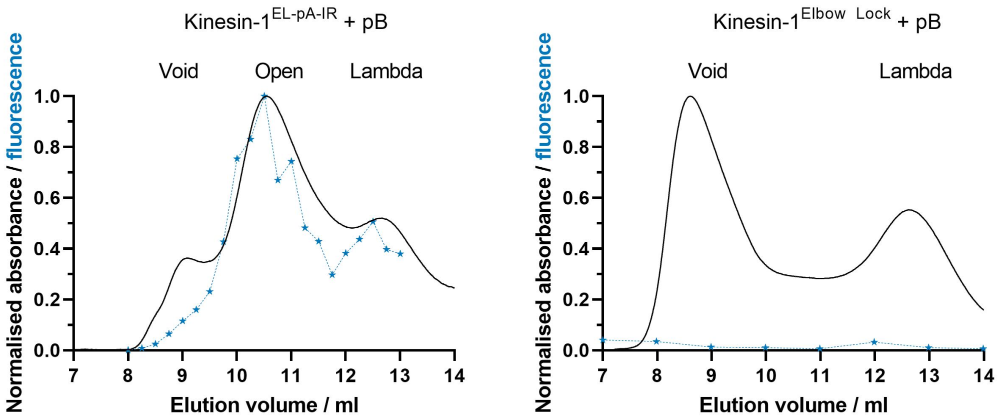

Fig. S8. Binding of peptide pB to Kinesin-1$^{\text{EL-pA-IR}}$ is observed by fluorescence in SEC (left) compared to no binding to Kinesin-1$^{\text{Elbow Lock}}$ (right). Peptide pB was added to purified Kinesin-1 (Kif5C, KLC1) protein at 4°C for 1h with agitation and then subject to SEC. Elution of protein in SEC was monitored by absorbance at 280 nm and normalised (black curve). Presence of peptide-B was monitored by fluorescence of TAMRA at 555 nm in individual fractions using a plate reader and normalised (blue stars). Coelution of peptide pB and Kinesin-1$^{\text{EL-pA-IR}}$ indicates binding.

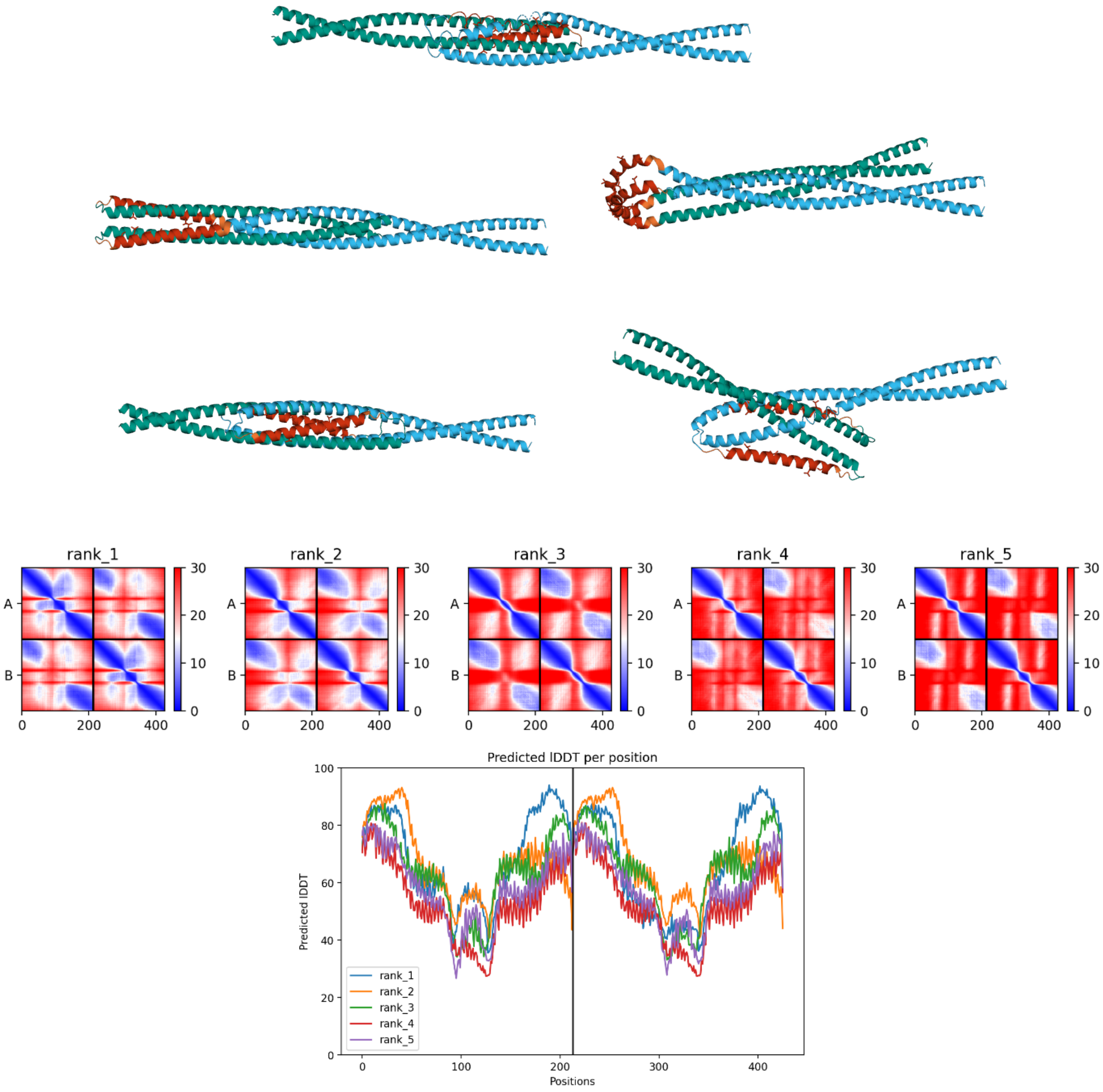

Sequence:

iskmksevkslvnrskqlesaqtdsnrkmnaserelaacqllisqheakiksltdymqnmeqkrrqle
esqdslseelaklraqekmhevsfqdkGGGQLEQELAALDQEIAAAEQELAALDWQIQGGGtrlqdae
evkkaleqqmeshreahqkqlsrlrdeieekqriideirdlnqklqleqerlssdynklkiedqerev
klekllll n

Fig. S9. AlphaFold2 models of Kinesin-1$^{EL\text{-}pA\text{-}Gly}$ CC2-elbow-CC3 with corresponding PAE and pLDDT plots and sequence. The five models vary but all have a folded structural prediction with a flexible hinge between CC2 (teal) and CC3 (cyan) with pA in red, and GGG linkers in yellow. Side chains are shown as sticks for the hydrophobic core of pA.

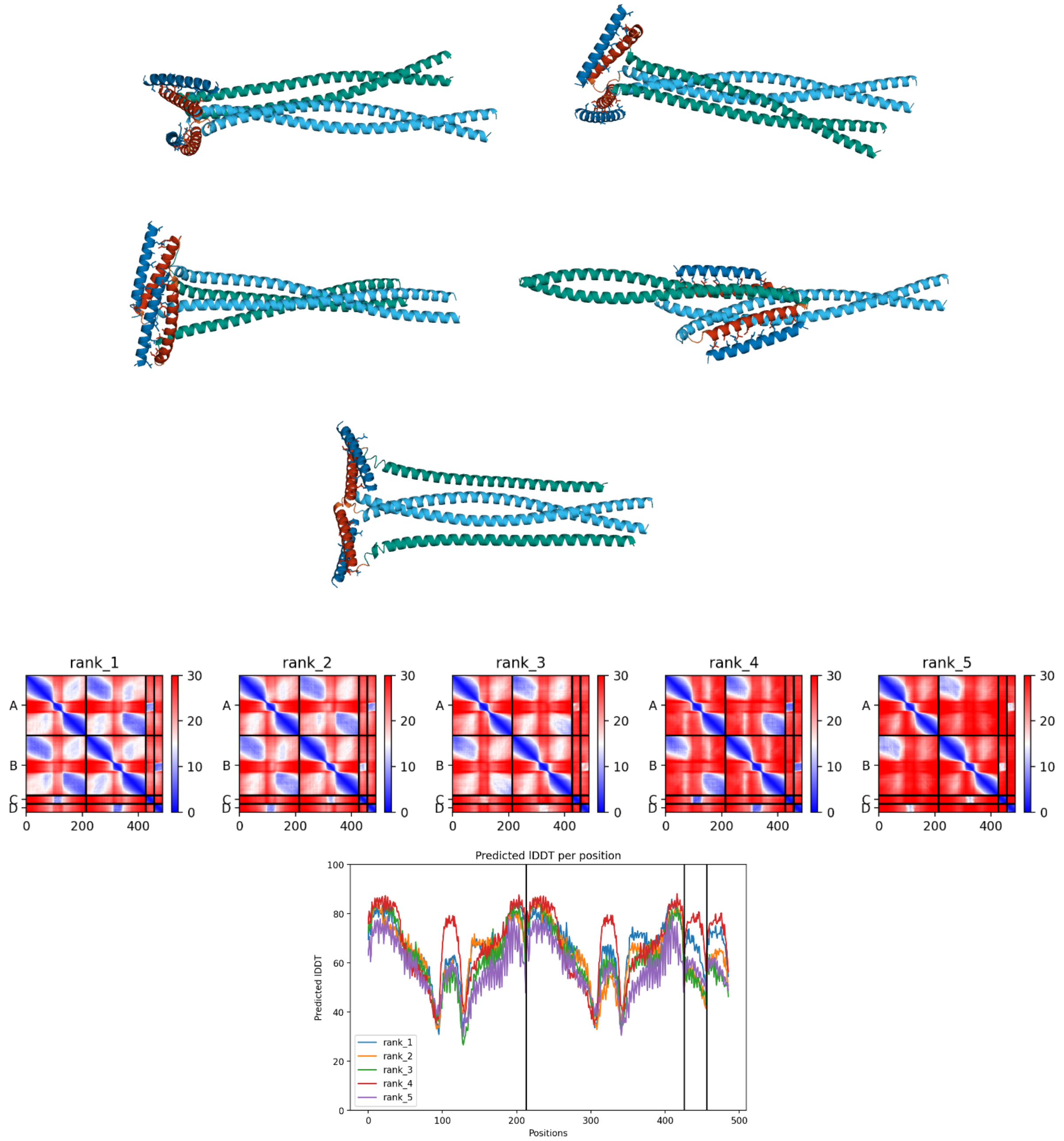

Sequence:

iskmksevkslvnrskqlesaqtdsnrkmnaserelaacqllisqheakiksltdymqnmeqkrrqle
esqdslseelaklraqekmhevsfqdkGGGQLEQELAALDQEIAAAEQELAALDWQIQGGGtrlqdae
evkkaleqqmeshreahqkqlsrlrdeieekqriideirdlnqklqleqerlssdynklkiedqerev
kleklllln

Peptide pB: GQLKQRRAALKQRIAALKQRRAALKWQIQG

Fig. S10. AlphaFold2 models of Kinesin-1$^{EL\text{-}pA\text{-}Gly}$ CC2-elbow-CC3 with peptide B with corresponding PAE and pLDDT plots and sequences The five models vary but all have a folded structural prediction with a flexible hinge between CC2 (teal) and CC3 (cyan) accommodating pA (red) binding to pB (blue). GGG linkers are in yellow and side chains are shown as sticks for the hydrophobic core of pA and pB.

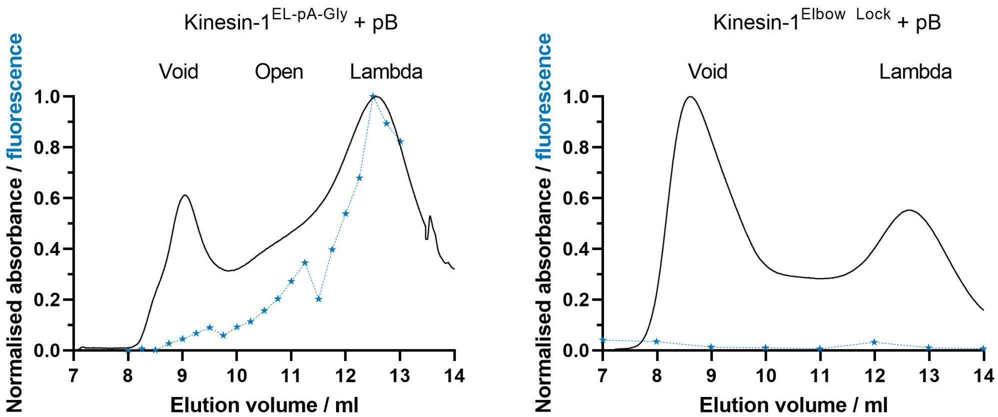

Fig. S11. Binding of peptide pB to Kinesin-1$^{\text{EL-pA-Gly}}$ is observed by fluorescence in SEC (left) compared to no binding to Kinesin-1$^{\text{Elbow Lock}}$ (right). Peptide pB was added to purified Kinesin-1 (Kif5C, KLC1) protein at 4°C for 1h with agitation and then subject to SEC. Elution of protein in SEC was monitored by absorbance at 280 nm and normalised (black curve). Presence of peptide-B was monitored by fluorescence of TAMRA at 555 nm in individual fractions using a plate reader and normalised (blue stars). Coelution of peptide pB and Kinesin-1$^{\text{EL-pA-Gly}}$ indicates binding.

Ranks 1-4

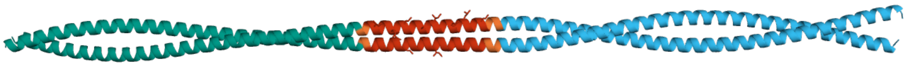

Ranks 5

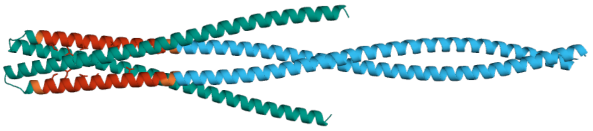

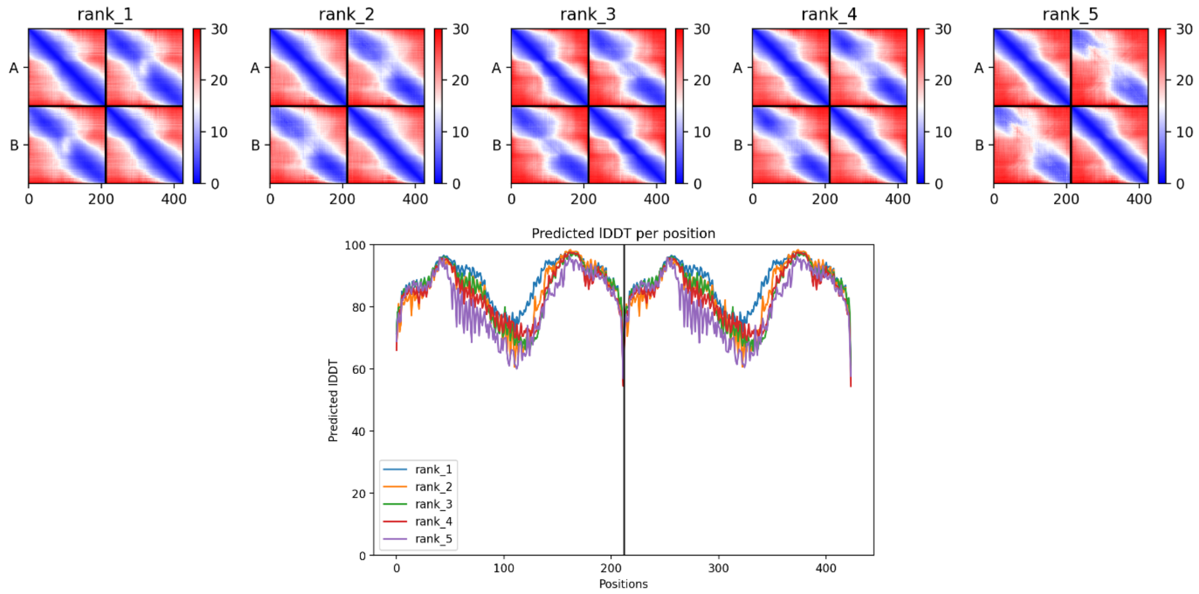

Sequence:

Iskmksevkslvnrskqlesaqtdsnrkmnaserelaacqllisqheakiksltdymqnmeqkrrqle
esqdslseelaklraqekmhevsfqdkAAAQLEQELAALDQEIAAAEQELAALDWQIQAAtrlqdaee
vkkaleqqmeshreahqkqlsrlrdeieekqriideirdlnqklqleqerlssdynklkiedqerevk
leklllln

Fig. S12. Alignment of AlphaFold2 models of Kinesin-1$^{EL\text{-}pA\text{-}Ala}$ CC2-elbow-CC3 with corresponding PAE and pLDDT plots and sequence. 4 aligned models (ranks 1-4) of Kinesin-1$^{EL\text{-}pA\text{-}Ala}$ CC2-elbow-CC3 closely overlay with the same extended coiled coil structural prediction of CC2 (teal) and CC3 (cyan) with peptide-A in red and AAA/AA linkers in yellow. The final model (rank 5) predicts a folded conformation. Side chains are shown as sticks for the hydrophobic core of pA.

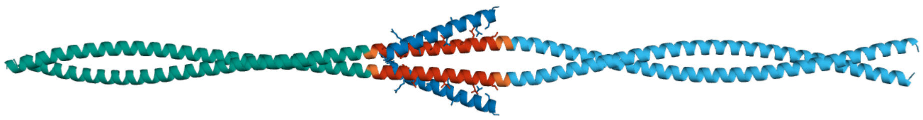

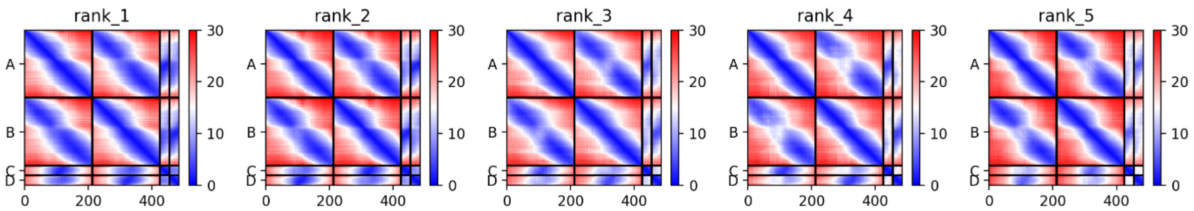

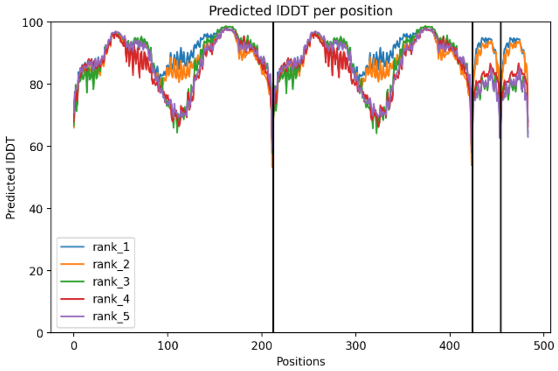

Sequence:

Iskmksevkslvnrskqlesaqtdsnrkmnaserelaacqllisqheakiksltdymqnmeqkrrqle
esqdslseelaklraqekmhevsfqdkAAAQLEQELAALDQEIAAAEQELAALDWQIQAAtrlqdaee
vkkaleqqmeshreahqkqlsrlrdeieekqriideirdlnqklqleqerlssdynklkiedqerevk
lekl llln

Peptide pB: GQLKQRRAALKQRIAALKQRRAALKWQIQG

Fig. S13. Alignment of AlphaFold2 models of Kinesin-1$^{EL\text{-}pA\text{-}Ala}$ CC2-elbow-CC3 with peptide B with corresponding PAE and pLDDT plots and sequences. All five models of Kinesin-1$^{EL\text{-}pA\text{-}IR}$ CC2-elbow-CC3 and peptide pB closely overlay with the same extended coiled coil structural prediction of CC2 (teal) and CC3 (cyan) with pA in red, peptide pB in blue and AAA/AA linkers in yellow. Side chains are shown as sticks for the hydrophobic core of pA and pB.

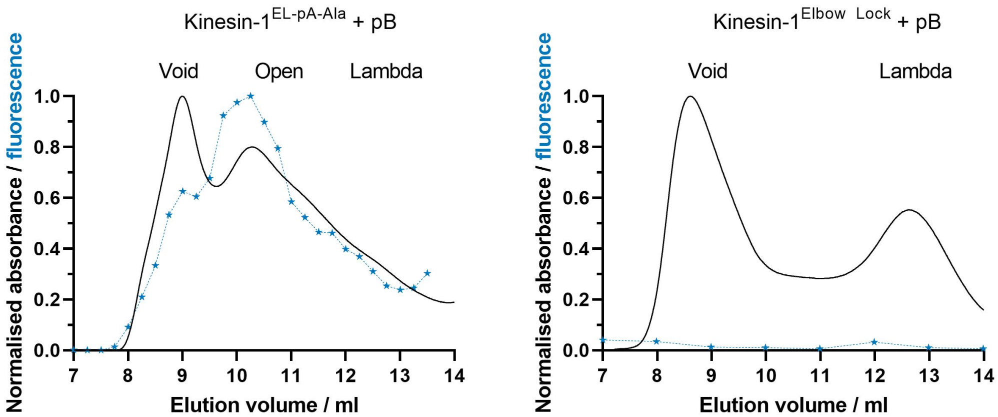

Fig. S14. Binding of peptide pB to Kinesin-1$^{EL-pA-Ala}$ is observed by fluorescence in SEC (left) compared to no binding to Kinesin-1$^{Elbow\ Lock}$ (right). Peptide-B was added to purified Kinesin-1 (Kif5C, KLC1) protein at 4ºC for 1h with agitation and then subject to SEC. Elution of protein in SEC was monitored by absorbance at 280 nm and normalised (black curve). Presence of peptide pB was monitored by fluorescence of TAMRA at 555 nm in individual fractions using a plate reader and normalised (blue stars). Coelution of peptide pB and Kinesin-1$^{EL-pA-Ala}$ indicates binding.

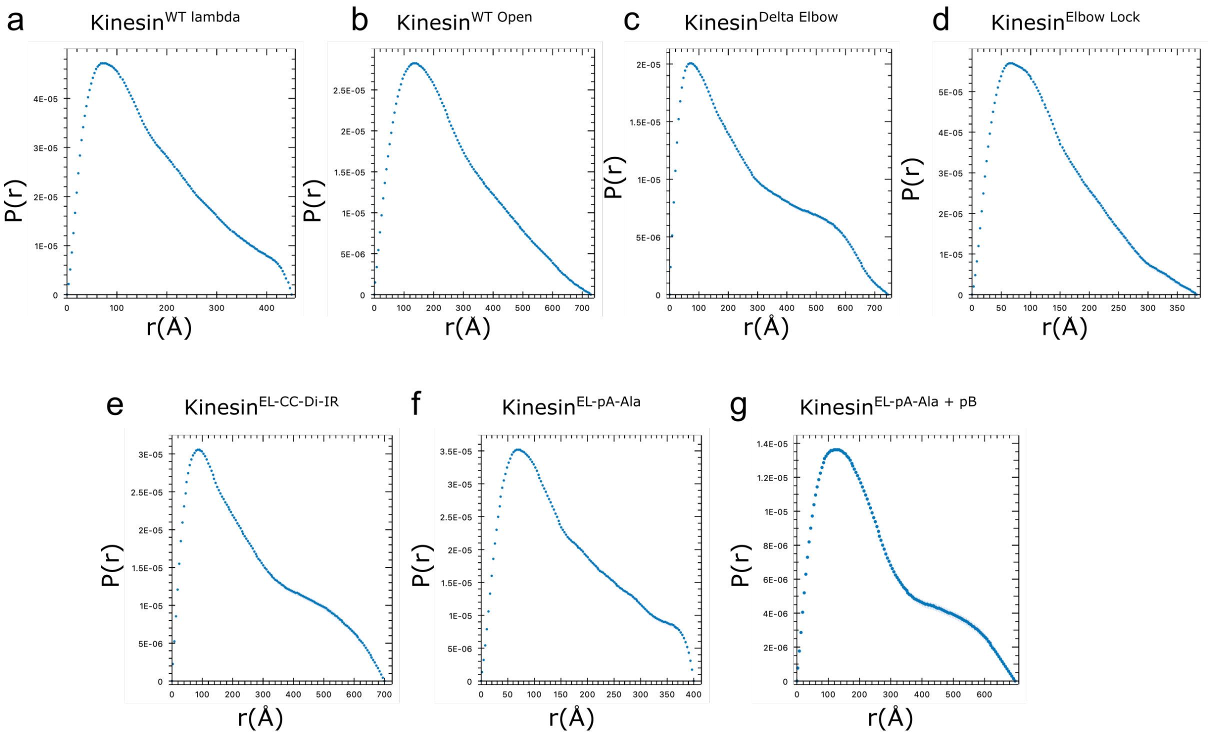

Fig. S15. Pair distance distribution plots (*P(r)*) for kinesin-1 variants from SEC-SAXS scattering data. a. Kinesin-1$^{WT\ Lambda}$, **b.** Kinesin-1$^{WT\ Open}$ **c.** Kinesin-1$^{Delta\ Elbow}$, **d.** Kinesin-1$^{Elbow\ Lock}$, **e.** Kinesin-1$^{EL-CC-Di-}$, **f.** Kinesin-1$^{EL-pA-Ala}$, **g.** Kinesin-1$^{EL-pA-Ala}$ + pB.

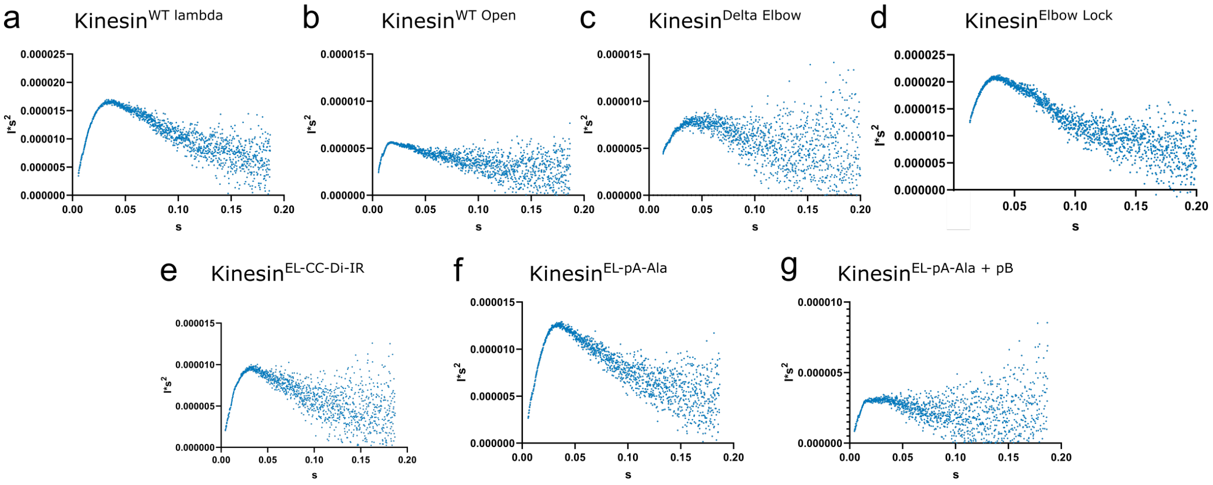

Fig. S16. Kratky plot indicated flexibility in all the kinesin-1 complexes from SEC-SAXS scattering data. Kratky plots give a qualitative assessment of globularity or flexibility in samples. Whilst globular proteins are expected to converge with a gaussian peak, these plots show a plateau at high s indicating flexibility in the complex. **a.** Kinesin-1$^{\mathrm{WT\ Lambda}}$, **b.** Kinesin-1$^{\mathrm{WT\ Open}}$ **c.** Kinesin-1$^{\mathrm{Delta\ Elbow}}$, **d.** Kinesin-1$^{\mathrm{Elbow\ Lock}}$, **e.** Kinesin-1$^{\mathrm{EL\text{-}CC\text{-}Di\text{-}}}$, **f.** Kinesin-1$^{\mathrm{EL\text{-}pA\text{-}Ala}}$, **g.** Kinesin-1$^{\mathrm{EL\text{-}pA\text{-}Ala\ +\ pB}}$.

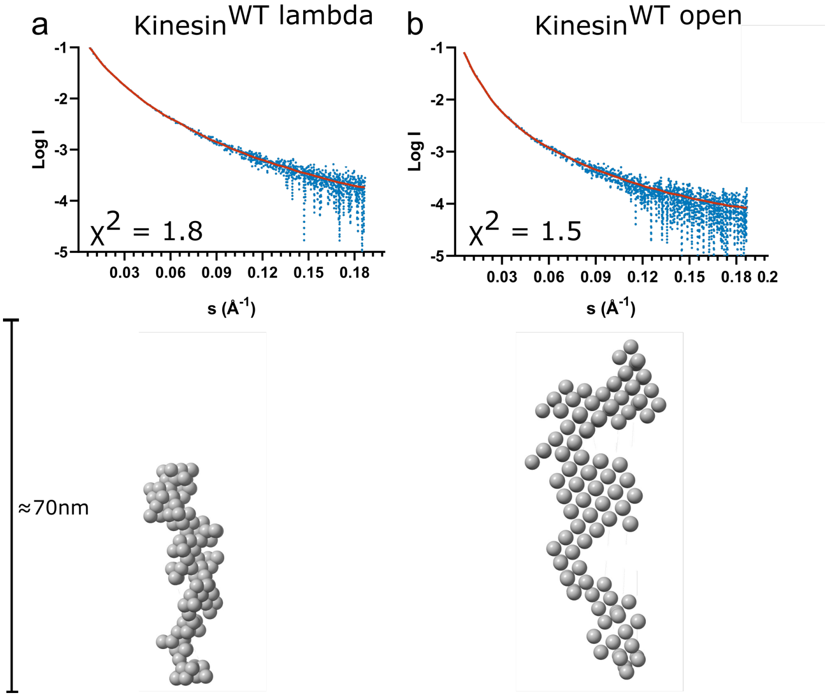

Fig. S17. SEC-SAXS in-solution shape determination and conformational states of Kinesin-1^{WT}. Top: Comparison of trimmed experimental scattering profiles (blue) with theoretical scattering profiles (red) generated from ab-initio models using DAMMIN. The quality of fit is assessed using the χ^2 statistic. Bottom: Representative models obtained from DAMMIN illustrating the shapes of the kinesin-1 variants, **a.** Kinesin-1$^{\mathrm{WT\ lambda}}$ is in the lambda state $D_{\max}$ = 45.0 nm χ^2=1.8, **b.** Kinesin-1$^{\mathrm{WT\ open}}$ is in the open state $D_{\max}$ = 72.9 nm χ^2 = 1.5.

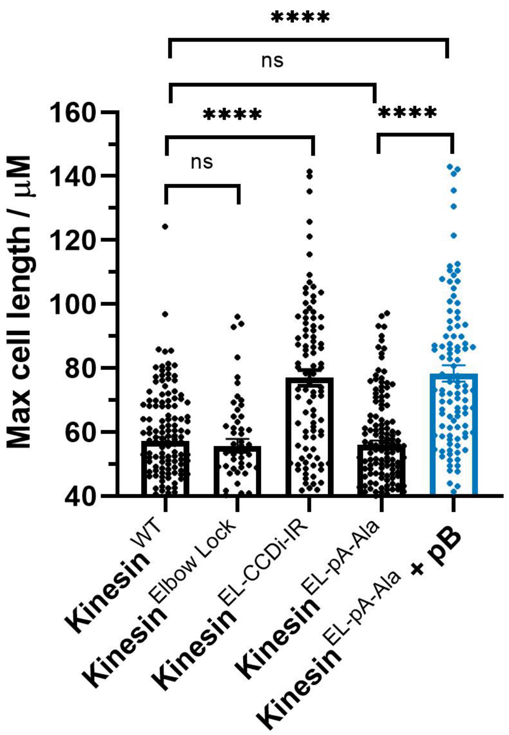

Fig. S18. Quantification of HeLa cell length in cells transfected with kinesin constructs. As in figure 4, HeLa cells were transfected with GFP-KLC2 and Ha-Kif5C (Kinesin-1^{WT}, Kinesin-$1^{Elbow\ Lock}$, Kinesin-$1^{EL\text{-}CC\text{-}Di\text{-}IR}$ or Kinesin-$1^{EL\text{-}pA\text{-}Ala}$) with (blue) /without (black) treatment with 2 μM TAMRA-labelled pB for 1 h. The maximum length of each transfected cell was measured for a minimum of 52 cells for each construct, pooled across 3 independent experiments. There is no significant difference between the length of cells transfected with Kinesin-1^{WT}, Kinesin-$1^{Elbow\ Lock}$ and Kinesin-$1^{EL\text{-}pA\text{-}Ala}$. By contrast, cells transfected with Kinesin-$1^{EL\text{-}CC\text{-}Di\text{-}IR}$ or Kinesin-$1^{EL\text{-}pA\text{-}Ala}$ and treated with peptide B are extended with a longer maximum length. Data are presented as individual values with bars showing mean ± S.E.M. and an unpaired two-tailed t-test is used for statistical analysis. ns: not significant ****: Kinesin-1^{WT} vs Kinesin-$1^{EL\text{-}pA\text{-}Ala + pB}$ $P = 1.87 \times 10^{-13}$, Kinesin-1^{WT} vs Kinesin-$1^{EL\text{-}CC\text{-}Di\text{-}IR}$ $P = 6.55 \times 10^{-13}$, Kinesin-$1^{EL\text{-}pA\text{-}Ala}$ vs Kinesin-$1^{EL\text{-}pA\text{-}Ala + pB}$ $P = 4.09 \times 10^{-15}$.

.
