# Supplementary material for: A de novo designed coiled coil-based switch regulates the microtubule motor kinesin-1
Source: Nat Chem Biol. 2024 Jun 7;20(7):916–23. doi: 10.1038/s41589-024-01640-2 (PMC11213707; doi:10.1038/s41589-024-01640-2)
Supplement: Supplementary file 2 — Reporting Summary [file 41589_2024_1640_MOESM2_ESM.pdf]

Corresponding author(s): Derek N Woolfson

Last updated by author(s): Apr 24, 2024

Reporting Summary

Nature Portfolio wishes to improve the reproducibility of the work that we publish. This form provides structure for consistency and transparency in reporting. For further information on Nature Portfolio policies, see our Editorial Policies and the Editorial Policy Checklist.

Statistics

For all statistical analyses, confirm that the following items are present in the figure legend, table legend, main text, or Methods section.

n/a	Confirmed	
☐	☒	The exact sample size (*n*) for each experimental group/condition, given as a discrete number and unit of measurement
☐	☒	A statement on whether measurements were taken from distinct samples or whether the same sample was measured repeatedly
☐	☒	The statistical test(s) used AND whether they are one- or two-sided<br>*Only common tests should be described solely by name; describe more complex techniques in the Methods section.*
☒	☐	A description of all covariates tested
☒	☐	A description of any assumptions or corrections, such as tests of normality and adjustment for multiple comparisons
☐	☒	A full description of the statistical parameters including central tendency (e.g. means) or other basic estimates (e.g. regression coefficient) AND variation (e.g. standard deviation) or associated estimates of uncertainty (e.g. confidence intervals)
☐	☒	For null hypothesis testing, the test statistic (e.g. *F*, *t*, *r*) with confidence intervals, effect sizes, degrees of freedom and *P* value noted<br>*Give P values as exact values whenever suitable.*
☒	☐	For Bayesian analysis, information on the choice of priors and Markov chain Monte Carlo settings
☒	☐	For hierarchical and complex designs, identification of the appropriate level for tests and full reporting of outcomes
☒	☐	Estimates of effect sizes (e.g. Cohen's *d*, Pearson's *r*), indicating how they were calculated

Our web collection on statistics for biologists contains articles on many of the points above.

Software and code

Policy information about availability of computer code

Data collection	AlphaFold2, SEC-SAXS data were collected at BL-21 at Diamond Light Source: Diamond light source, UK (https:// www.diamond.ac.uk/).
Data analysis	SEC-SAXS: ATSAS-3.2.1-2: CHROMIXS, PRIMUSQT, GNOM, DAMMIN. Graph plotting: Prism 9 Image Analysis: ImageJ2 (2.9.0/1.3t), Cartoon generation: PYMOL 2.1

For manuscripts utilizing custom algorithms or software that are central to the research but not yet described in published literature, software must be made available to editors and reviewers. We strongly encourage code deposition in a community repository (e.g. GitHub). See the Nature Portfolio guidelines for submitting code & software for further information.

Data

Policy information about availability of data

All manuscripts must include a data availability statement. This statement should provide the following information, where applicable:

- Accession codes, unique identifiers, or web links for publicly available datasets
- A description of any restrictions on data availability
- For clinical datasets or third party data, please ensure that the statement adheres to our policy

All data needed to interpret, verify, and extend the results presented in this article are available in the manuscript, supplementary information, and source files. In addition, the raw data used in this publication has been deposited in the Zenodo repository DOI: 10.5281/zenodo.11061566.

Human research participants

Policy information about studies involving human research participants and Sex and Gender in Research.

Reporting on sex and gender	N/A
Population characteristics	N/A
Recruitment	N/A
Ethics oversight	N/A

Note that full information on the approval of the study protocol must also be provided in the manuscript.

Field-specific reporting

Please select the one below that is the best fit for your research. If you are not sure, read the appropriate sections before making your selection.

☒ Life sciences ☐ Behavioural & social sciences ☐ Ecological, evolutionary & environmental sciences

For a reference copy of the document with all sections, see nature.com/documents/nr-reporting-summary-flat.pdf

Life sciences study design

All studies must disclose on these points even when the disclosure is negative.

Sample size	Biophysical measurements do not have sample sizes but SEC experiments were validated by replication three times. For comparison of particle length in NS-EM, 100 particles were measured in each condition and gave P<0.0001. For quantification of cell phenotypes, the experiments were repeated three times and a minimum of 45 cells measured, pooled from three experiments, giving significant differences between conditions (P = 0.0237 - P = 4.09 x 10-15). This sample size is consistent with previous studies, e.g https://doi.org/10.1038/s41589-022-01076-6
Data exclusions	No data were excluded from this study.
Replication	All attempts at replication of the experiments in this study were successful, and mean and variance values were generated from at least 3 independent measurements in all cases.
Randomization	This study did not involve samples being allocated into experimental groups, and therefore statistical hypothesis issues related to randomisation do not apply to this study.
Blinding	This study does not involve experiments where the outcome would be influenced by blinding, and therefore statistical hypothesis issues related to blinding do not apply to this study.

Reporting for specific materials, systems and methods

We require information from authors about some types of materials, experimental systems and methods used in many studies. Here, indicate whether each material, system or method listed is relevant to your study. If you are not sure if a list item applies to your research, read the appropriate section before selecting a response.

Materials & experimental systems

n/a	Involved in the study	
☐	☒	Antibodies
☐	☒	Eukaryotic cell lines
☒	☐	Palaeontology and archaeology
☒	☐	Animals and other organisms
☒	☐	Clinical data
☒	☐	Dual use research of concern

Methods

n/a	Involved in the study	
☒	☐	ChIP-seq
☒	☐	Flow cytometry
☒	☐	MRI-based neuroimaging

Antibodies

Antibodies used	Anti-HA antibody, Mouse monoclonal, clone HA-7, purified from hybridoma cell culture, Sigma Aldrich H3663. Goat anti-Mouse IgG Secondary Antibody, Alexa Fluor™ 647 (A-21235 Thermo Fisher) .
Validation	Monoclonal Anti-HA, Clone HA-7 (mouse IgG1 isotype) is derived from the HA-7 hybridoma produced by the fusion of mouse myeloma cells and splenocytes from a BALB/c mouse immunized with a synthetic peptide corresponding to amino acid residues YPYDVPDYA (98-106) of the human influenza virus hemagglutinin (HA), conjugated to KLH. The isotype is determined by a double diffusion immunoassay using Mouse Monoclonal Antibody Isotyping Reagents (Cat. No. ISO2). The antibody recognizes an epitope located within the sequence YPYDVPDYA (residues 98-106) of the human influenza virus hemagglutinin, known as the HA tag. The product is reactive with HA-tagged fusion proteins expressed at either the amino or the carboxy terminus of the fusion protein. Applications include ELISA, immunoblotting, immunocytochemistry, and immunoprecipitation. The antibody has been validated by the manufacturer and cited in several publications available in the Sigma-Aldrich Product Information Sheet- H3663.The antibody was validated in this study for immunofluorescence at a dilution of 1/1000 as it gives a clean staining pattern, consistent with the literature and with minimal background signal in the absence of an Ha tag.

Eukaryotic cell lines

Policy information about cell lines and Sex and Gender in Research

Cell line source(s)	HeLa cells, ATCC CCL2
Authentication	HeLa cells were sent to Eurofins Genomics on 08.07.2021 for a cell line authentication test with comparison with online DSMZ database and a certificate issued. Method: DNA isolation was carried out from cell pellet (cell layer).<br>Genetic characteristics were determined by PCR-single-locus-technology. 16 independent PCR-systems D8S1179, D21S11, D7S820, CSF1PO, D3S1358, TH01, D13S317, D16S539, D2S1338, AMEL, D5S818, FGA, D19S433, vWA, TPOX and D18S51 were investigated. In parallel, positive and negative controls were carried out yielding correct results.
Mycoplasma contamination	Cell lines were monitored for Mycoplasma contamination through routine DAPI staining and tested negative for mycoplasma contamination.
Commonly misidentified lines<br>(See ICLAC register)	No commonly misidentified cell lines were used in this study.
